# Supplementary figures and images for: Population structure and diversity of common bean (Phaseolus vulgaris L.) landraces in the Peruvian Amazon
Source: PLoS One. 2026 Jul 20;21(7):e0332680. doi: 10.1371/journal.pone.0332680 (PMC13384298; doi:10.1371/journal.pone.0332680)

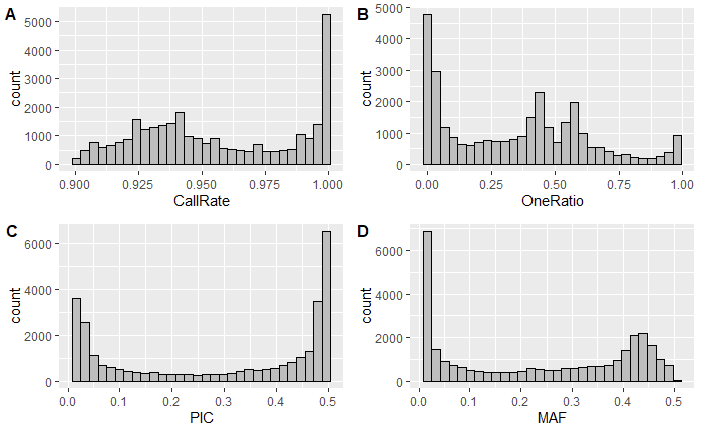

Supplement: S1 Fig — A) Callrate, B) OneRatio, C) Polymorphic Index Content (PIC), and E) minimum allele frequency (MAF) on the initial filtered ser 29865 SNP markers. (TIFF) [file pone.0332680.s001.tiff]

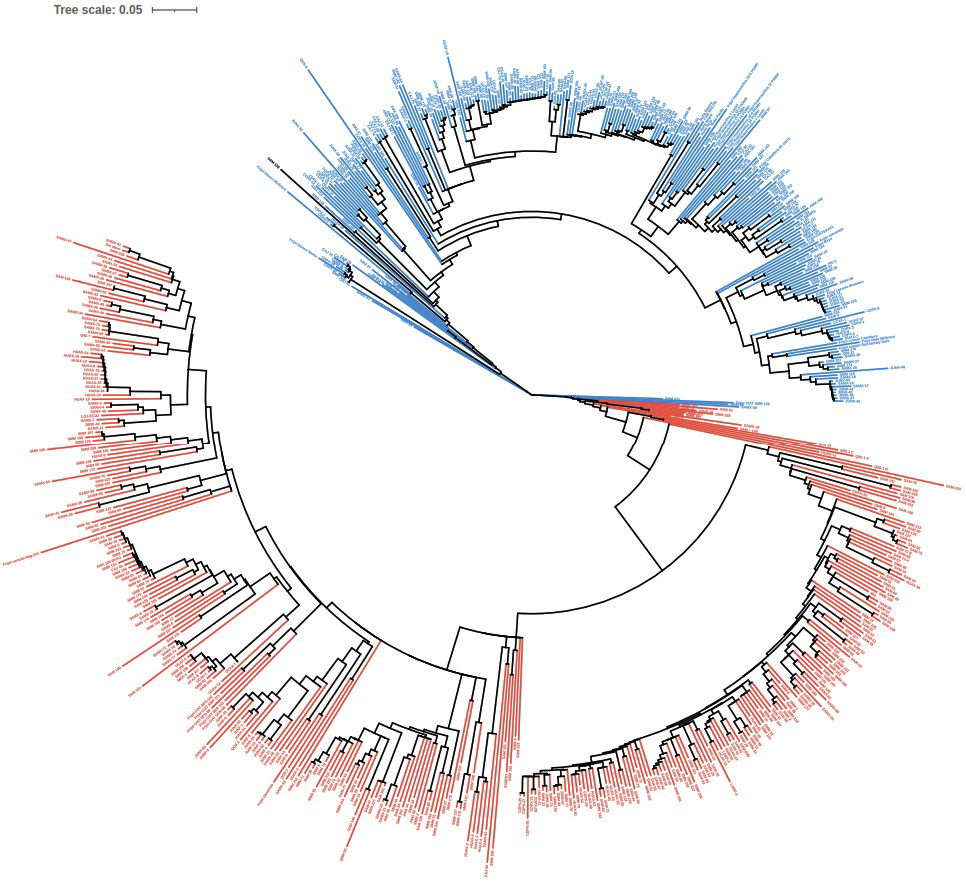

Supplement: S2 Fig — (TIFF) [file pone.0332680.s002.tiff]

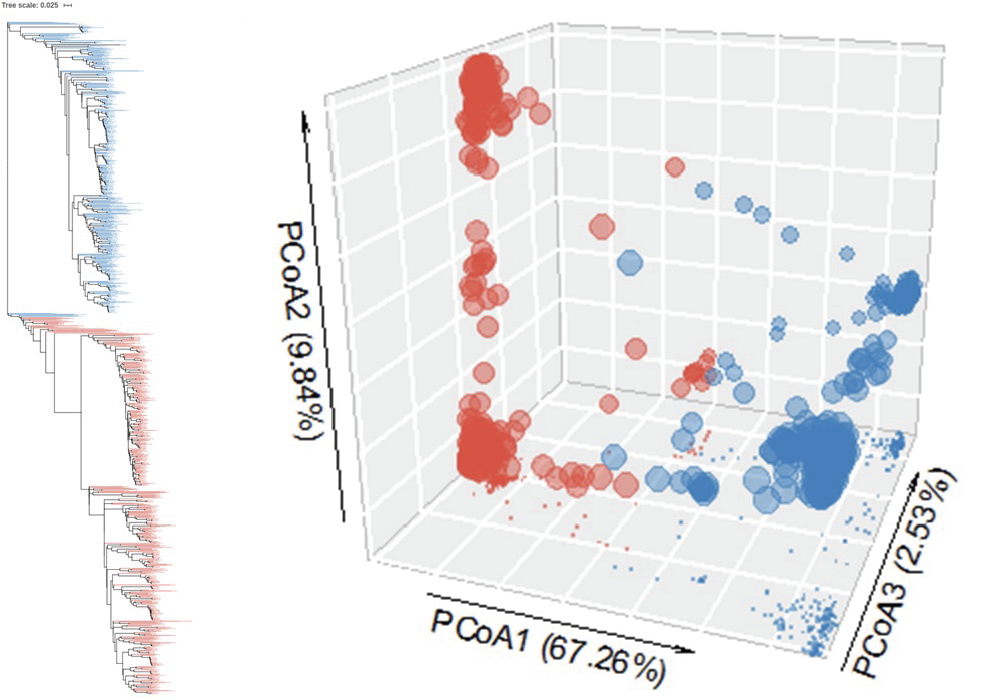

Supplement: S3 Fig — (TIFF) [file pone.0332680.s003.tiff]

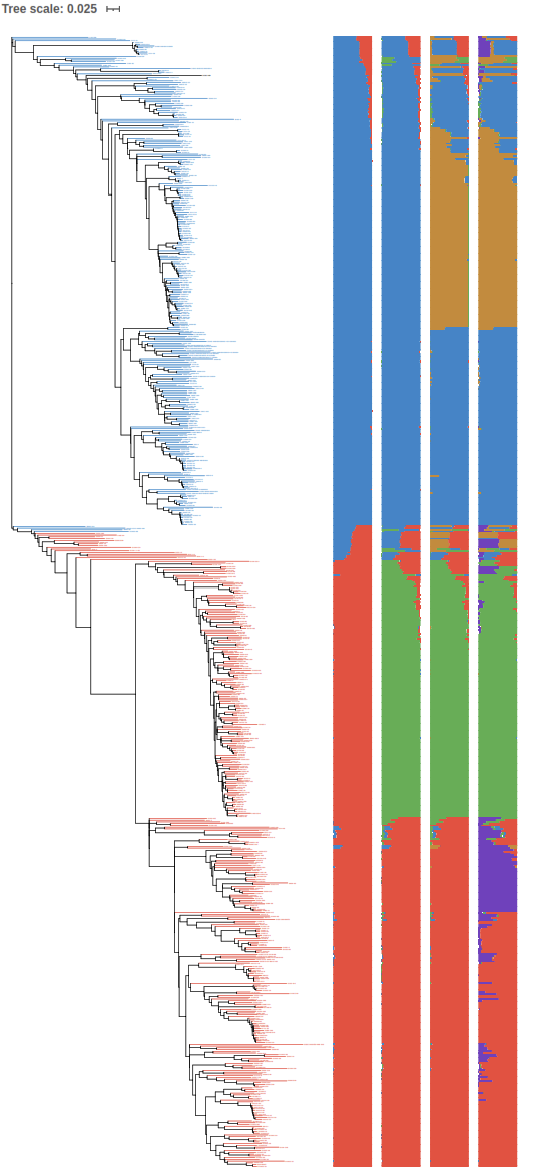

Supplement: S4 Fig — K2 to K5 (from left to right). (TIFF) [file pone.0332680.s004.tiff]
