# Supplementary material for: Population structure and diversity of common bean (Phaseolus vulgaris L.) landraces in the Peruvian Amazon
Source: PLoS One. 2026 Jul 20;21(7):e0332680. doi: 10.1371/journal.pone.0332680 (PMC13384298; doi:10.1371/journal.pone.0332680)
Supplement: S1 File — (DOCX) [file pone.0332680.s005.docx]

**Morphological characterization data of common bean_Amazonia_Peru**

**DraftUnpublished**

Blas, Raul, 2026, "Morphological characterization data of common bean_Amazonia_Peru", <https://doi.org/10.17026/LS/6IRCBV>, DANS Data Station Life Sciences, DRAFT VERSION

Learn about [Data Citation Standards](https://dataverse.org/best-practices/data-citation).

**DArTseq_SNP_647x23050 markers_common_bean_Peru**

**DraftUnpublished**

Blas, Raul, 2026, "DArTseq_SNP_647x23050 markers_common_bean_Peru", <https://doi.org/10.17026/LS/SWIWOJ>, DANS Data Station Life Sciences, DRAFT VERSION

Learn about [Data Citation Standards](https://dataverse.org/best-practices/data-citation).
