# Supplementary material for: Population structure and diversity of common bean (Phaseolus vulgaris L.) landraces in the Peruvian Amazon
Source: PLoS One. 2026 Jul 20;21(7):e0332680. doi: 10.1371/journal.pone.0332680 (PMC13384298; doi:10.1371/journal.pone.0332680)
Supplement: Attachment — Submitted filename: PONE-D-25-47793_R1_reviewer.pdf [file pone.0332680.s008.pdf]

PLOS One

Population structure and diversity of common bean (Phaseolus vulgaris L.) landraces in the Peruvian Amazon

--Manuscript Draft--

**Manuscript Number:**	PONE-D-25-47793R1
**Article Type:**	Research Article
**Full Title:**	Population structure and diversity of common bean (Phaseolus vulgaris L.) landraces in the Peruvian Amazon
**Short Title:**	Common bean from Peruvian Amazon
**Corresponding Author:**	Rodomiro Ortiz, PhD<br>SLU: Sveriges lantbruksuniversitet<br>Alnarp, Skåne SWEDEN
**Keywords:**	Amazonian landraces; common bean; genetic diversity; Morphological characterization; Phaseolus vulgaris; plant genetic resources; SNP markers
**Abstract:**	Common bean (Phaseolus vulgaris L.) is a globally important legume with significant nutritional and agronomic value. Genetic diversity in Amazonian landraces remains undercharacterized, limiting their utilization in breeding programs. Here we analyzed 647 accessions from the Peruvian Amazon using 18 morphological traits and 23,050 high-quality DArTseq single nucleotide polymorphisms (SNPs). Our results revealed two major genetic groups corresponding to Andean and Mesoamerican gene pools, each subdivided into distinct subgroups, exhibiting high allelic richness and structured diversity. Despite abundant polymorphic loci, observed heterozygosity was low, consistent with the species' self-pollinating nature. These findings highlight the Peruvian Amazon as a reservoir of unique genetic variation, underscoring the need for integrated in situ and ex situ conservation strategies. This germplasm offers valuable resources for breeding programs targeting tropical adaptation and resilience, contributing to sustainable agriculture and food security.
**Order of Authors:**	Jorge Tobaru
	Marvin Barrera-Lozano
	Carlos D. Vecco-Giove
	Jorge Peláez
	Mack Pinchi
	Yesenia Vargas
	Jorge Chuquillanqui
	Julian Soto-Torres
	Ronald Robles
	David Saravia
	Cesar Petroli
	Jorge D. Etchevers
	Rodomiro Ortiz, PhD
	Raul Blas, PhD
**Response to Reviewers:**	The reply to reviewers using a point to point writing style included among files uploaded.
**Additional Information:**	
**Question**	**Response**
**Financial Disclosure**	Yes

Powered by Editorial Manager® and ProduXion Manager® from Aries Systems Corporation

Enter a financial disclosure statement that describes the sources of funding for the work included in this submission. Review the submission guidelines for detailed requirements. View published research articles from *PLOS ONE* for specific examples.<br><br>This statement is required for submission and **will appear in the published article** if the submission is accepted. Please make sure it is accurate.<br><br>**Funded studies**<br>Enter a statement with the following details:<br>• Initials of the authors who received each award<br>• Grant numbers awarded to each author<br>• The full name of each funder<br>• URL of each funder website<br>• Did the sponsors or funders play any role in the study design, data collection and analysis, decision to publish, or preparation of the manuscript?<br><br>Did you receive funding for this work?	
Please add funding details.<br>as follow-up to "**Financial Disclosure**<br><br>Enter a financial disclosure statement that describes the sources of funding for the work included in this submission. Review the submission guidelines for detailed requirements. View published research articles from *PLOS ONE* for specific examples.<br><br>This statement is required for submission and **will appear in the published article** if the submission is accepted. Please make sure it is accurate.	This work was supported, in whole by Peruvian government funding-PROCIENCIA, E041 - Proyectos de Investigación Básica y Aplicada (PE501079155-2022), through the “Genómica del frijol Phaseolus spp. asociada con la diversidad cultural y ecosistémica en la Amazonia peruana” project.

Powered by Editorial Manager® and ProduXion Manager® from Aries Systems Corporation

**Funded studies**<br>Enter a statement with the following details:<br>• Initials of the authors who received each award<br>• Grant numbers awarded to each author<br>• The full name of each funder<br>• URL of each funder website<br>• Did the sponsors or funders play any role in the study design, data collection and analysis, decision to publish, or preparation of the manuscript?<br><br>Did you receive funding for this work?"	
Please select the country of your main research funder (please select carefully as in some cases this is used in fee calculation).<br>as follow-up to "**Financial Disclosure**<br><br>Enter a financial disclosure statement that describes the sources of funding for the work included in this submission. Review the submission guidelines for detailed requirements. View published research articles from *PLOS ONE* for specific examples.<br><br>This statement is required for submission and **will appear in the published article** if the submission is accepted. Please make sure it is accurate.<br><br>**Funded studies**<br>Enter a statement with the following details:<br>• Initials of the authors who received each award<br>• Grant numbers awarded to each author<br>• The full name of each funder<br>• URL of each funder website<br>• Did the sponsors or funders play any role in the study design, data collection and analysis, decision to publish, or preparation of the manuscript?<br><br>Did you receive funding for this work?"	PERU - PE
**Competing Interests**	The authors have declared that no competing interests exist.

Use the instructions below to enter a competing interest statement for this submission. On behalf of all authors, disclose any competing interests that could be perceived to bias this work—acknowledging all financial support and any other relevant financial or non-financial competing interests.<br><br>This statement is required for submission and **will appear in the published article** if the submission is accepted. Please make sure it is accurate and that any funding sources listed in your Funding Information later in the submission form are also declared in your Financial Disclosure statement.<br><br>View published research articles from *PLOS ONE* for specific examples.<br><br>**NO authors have competing interests**<br><br>Enter: *The authors have declared that no competing interests exist.*<br><br>**Authors with competing interests**<br><br>Enter competing interest details beginning with this statement:<br><br>*I have read the journal's policy and the authors of this manuscript have the following competing interests: [insert competing interests here]*<br><br>* typeset	
**Ethics Statement**<br><br>Enter an ethics statement for this submission. This statement is required if the study involved:<br><br>• Human participants<br>• Human specimens or tissue<br>• Vertebrate animals or cephalopods<br>• Vertebrate embryos or tissues	This article does not contain any studies with human participants and animals and ethical approval is not applicable.

Powered by Editorial Manager® and ProduXion Manager® from Aries Systems Corporation

• Field research<br><br>Write "N/A" if the submission does not require an ethics statement.<br><br>General guidance is provided below. Consult the submission guidelines for detailed instructions. **Make sure that all information entered here is included in the Methods section of the manuscript.**	

Powered by Editorial Manager® and ProduXion Manager® from Aries Systems Corporation

**Format for specific study types**<br><br>**Human Subject Research (involving human participants and/or tissue)**<br>• Give the name of the institutional review board or ethics committee that approved the study<br>• Include the approval number and/or a statement indicating approval of this research<br>• Indicate the form of consent obtained (written/oral) or the reason that consent was not obtained (e.g. the data were analyzed anonymously)<br><br>**Animal Research (involving vertebrate animals, embryos or tissues)**<br>• Provide the name of the Institutional Animal Care and Use Committee (IACUC) or other relevant ethics board that reviewed the study protocol, and indicate whether they approved this research or granted a formal waiver of ethical approval<br>• Include an approval number if one was obtained<br>• If the study involved *non-human primates*, add *additional details* about animal welfare and steps taken to ameliorate suffering<br>• If anesthesia, euthanasia, or any kind of animal sacrifice is part of the study, include briefly which substances and/or methods were applied<br><br>**Field Research**<br><br>Include the following details if this study involves the collection of plant, animal, or other materials from a natural setting:<br>• Field permit number<br>• Name of the institution or relevant body that granted permission	
**Data Availability**<br><br>Authors are required to make all data underlying the findings described fully available, without restriction, and from the time of publication. PLOS allows rare exceptions to address legal and ethical concerns. See the PLOS Data Policy and FAQ for detailed information.	Yes - all data are fully available without restriction

Powered by Editorial Manager® and ProduXion Manager® from Aries Systems Corporation

A Data Availability Statement describing where the data can be found is required at submission. Your answers to this question constitute the Data Availability Statement and **will be published in the article**, if accepted.<br><br>**Important:** Stating 'data available on request from the author' is not sufficient. If your data are only available upon request, select 'No' for the first question and explain your exceptional situation in the text box.<br><br>Do the authors confirm that all data underlying the findings described in their manuscript are fully available without restriction?	
**Describe where the data may be found in full sentences. If you are copying our sample text, replace any instances of XXX with the appropriate details.**<br><br>• If the data are **held or will be held in a public repository**, include URLs, accession numbers or DOIs. If this information will only be available after acceptance, indicate this by ticking the box below. For example: *All XXX files are available from the XXX database (accession number(s) XXX, XXX.*).<br>• If the data are all contained **within the manuscript and/or Supporting Information files**, enter the following: *All relevant data are within the manuscript and its Supporting Information files.*<br>• If neither of these applies but you are able to provide **details of access elsewhere**, with or without limitations, please do so. For example:<br><br>*Data cannot be shared publicly because of [XXX]. Data are available from the XXX Institutional Data Access / Ethics Committee (contact via XXX) for researchers who meet the criteria for access to confidential data.*<br><br>*The data underlying the results presented in the study are available from (include the name of the third party*	Blas, Raul, 2026, "Morphological characterization data of common bean_Amazonia_Peru", https://doi.org/10.17026/LS/6IRCBV, DANS Data Station Life Sciences, DRAFT VERSION<br><br>Blas, Raul, 2026, "DArTseq_SNP_647x23050 markers_common_bean_Peru", https://doi.org/10.17026/LS/SWIWOJ, DANS Data Station Life Sciences, DRAFT VERSION

and contact information or URL).<br>• This text is appropriate if the data are owned by a third party and authors do not have permission to share the data.<br><br>* typeset	
Additional data availability information:	Tick here if the URLs/accession numbers/DOIs will be available only after acceptance of the manuscript for publication so that we can ensure their inclusion before publication.

Manuscript Click here to access/download;Manuscript;Manuscript.docx

Population structure and diversity of common bean (*Phaseolus vulgaris* L.) landraces in the Peruvian Amazon

Jorge Tobaru[1], Marvin Barrera-Lozano[2], Carlos D. Vecco-Giove[2], Jorge Peláez[2], Mack Pinchi[3], Yesenia Vargas[1], Jorge Chuquillanqui[1], Julian Soto-Torres[4], Ronald Robles[4], David Saravia[5], Cesar Petroli[6], Jorge D. Etchevers[7], Rodomiro Ortiz[8] & Raul Blas[1]

[1] Universidad Nacional Agraria La Molina (UNALM), Facultad de Agronomía, Programa de Leguminos de Grano y Oleaginosas. La Molina-Lima, Perú

[2] , Universidad Nacional de San Martín, Facultad de Ciencias Agrarias (UNSM). Tarapoto-San Martín, Perú

[3] Universidad Nacional de Ucayali (UNU), Facultad de Ciencias Agropecuarias. Pucallpa-Ucayali, Perú

[4] International Potato Center (CIP), La Molina, Lima- Peru

[5] Instituto Nacional de Innovación Agraria – INIA, La Molina, Lima- Peru

[6] International Maize and Wheat Improvement Center (CIMMYT), El Batán, Texcoco, Mexico
[7] Colegio de Postgraduados, Texcoco-Mexico

[8] Swedish University of Agricultural Sciences (SLU), Department of Plant Breeding, Alnarp, Sweden

Abstract

Common bean (*Phaseolus vulgaris* L.) is a worldwide important legume with significant nutritional and agronomic value. Despite its global importance, the genetic diversity in Amazonian landraces remains undercharacterized, limiting their utilization in breeding programs. Accordingly, the objective of this study was to comprehensively evaluate the genetic diversity and population structure of common bean populations from the Peruvian Amazon by integrating phenotypic and genomic approaches. A total of 476 accessions were characterized using 18 standardized agro-morphological traits, while a broader set of 647 accessions was analyzed using 23,050 high-quality DArTseq single nucleotide polymorphism (SNP) markers. Our results revealed two major genetic groups corresponding to Andean and Mesoamerican gene pools, each subdivided into distinct subgroups, exhibiting high allelic richness and structured diversity. Despite abundant polymorphic loci, observed heterozygosity was low, consistent with the species' self-pollinating nature. These findings highlight the Peruvian Amazon as a reservoir of unique genetic variation, underscoring the need for integrated in situ and ex situ conservation strategies. This germplasm offers valuable resources for breeding programs targeting tropical adaptation and resilience, contributing to sustainable agriculture and food security.

Keywords: Amazonian landraces, common bean, genetic diversity, morphological characterization, *Phaseolus vulgaris*, plant genetic resources, SNP markers

Introduction

The common bean (*Phaseolus vulgaris* L.) an annual leguminous self-pollinated crop is the most widely cultivated species of the genus Phaseolus and a major source of dietary vegetal protein and income worldwide; which is a diploid (2n = 2x = 22) species and its genome size according to the first telomere-to-telomere (T2T) genome assembly was 560.30 Mb and predicted 29,925 protein-coding genes [1] . The origin of *P. vulgaris* remains controversial, separating it from Mesoamerica and the Andean region. However, recent research using molecular markers, including SNPs, shows that this species' origin is Mesoamerica [2] [3] [4] [5]. Genetic and phylogeographic evidence indicates three distinct wild gene pools: 1) Mesoamerica (Mexico, Central America, Colombia and Venezuela), 2) the Andes (southern Peru, Bolivia and Argentina), and 3) northern Peru/Ecuador; nevertheless, the three common bean genetic groups belong to the primary gene pool [6]. From those, two main domesticated groups emerged belonging to the Mesoamerican and Andean gene pools, this domestication process has been done independently in the Andes and Mesoamerica, which are associated with two Andean and Mesoamerican gen pools, respectively [7] [8]. Both types of beans were introduced to other continents such as Europe, Africa, and Asia, where their widespread acceptance has led to debate about whether Europe can be a secondary center of diversification [9].

At the present, it is a staple crop in many parts of the world, most widely cultivated legume species worldwide, mainly due to its nutritional composition, being rich in amino acids, minerals, and vitamins—especially lysine, iron, zinc and folic acid [10]; presents medicinal attributes such as the prevention of cardiovascular diseases [11]; and has an ecological importance, it fixes atmospheric nitrogen reducing all the environmental costs involved in the acquisition of chemical fertilizers [12]. For this reason, the United Nations declared in 2016 as "the international year of legumes" in its 68th session [13], the common bean being within the legumes, considered one of the essential crops to promote food security. Common bean has thus become an important source of income and nutrients for small farmers in the Caribbean, Latin America, Asia, and Africa, where account for 77% of global production [14] [15] [16]. In the case of Brazil, a predominantly Amazonian region, the Mesoamerican gene pool is found in a greater proportion (79%) and the Andean gene pool in a lesser proportion (21%) [4].

In Peru, the common bean is cultivated across diverse agroecological zones, ranging from coastal valleys to highland and Amazonian regions. According to national statistics [17], production covers approximately 42,575 hectares annually, with San Martín, Piura, and Cajamarca being leading regions; with around 14% of this area located in the Amazon region—2.0% less than the area recorded in 2023. Beyond its economic relevance, the crop plays a crucial role in household nutrition and local food security. Despite its importance, most studies on common bean in Peru have focused on commercial cultivars grown in the highlands and coastal regions, emphasizing agronomic practices and genetic improvement using both local and introduced cultivars. Conversely, there is limited research conducted on the cultivars grown by farmers in indigenous communities of the Peruvian Amazon rainforest. According to the Genesys database, from Peru holds 3,497 accessions of common beans, of which 2,838 (81%) are classified as traditional cultivars, 516 (15%) are results from breeding programs, and 143 (4%) correspond to wild or near-wild forms [18]. The International Center for Tropical Agriculture (CIAT) holds the global reference collection, while the Peruvian National Institute for Agrarian Innovation (INIA) maintains the national bean germplasm bank, which

officially has 58 accessions of beans and 146 accessions of popping bean mainly from the Andean region [19] [20]. Despite these efforts, landraces and wild relatives from the Peruvian Amazon remain underrepresented, leaving a gap in the documentation of local agrobiodiversity.

Research on the diversity of cultivated common bean in Peru remains limited and has largely focused on specific bean types or restricted geographic areas. Most previous studies have emphasized popping beans (ñuña, in the Quechua language), employing phenotypic and molecular markers to characterize their diversity and differentiation [21,22], or have concentrated on isolated localities rather than addressing diversity at a broader regional scale [23]. In the central Peruvian Amazon, cultivated and sociocultural diversity of common bean has been documented, highlighting the role of Indigenous communities and traditional knowledge in shaping bean diversity through local management practices and cultural preferences [24]. More recently, agromorphological characterization of 58 Phaseolus spp. accessions collected in the Amazonas region identified several promising genotypes, with grain yields exceeding 2.77 t ha^{-1}, underscoring their potential value for use in breeding programs [25]. However, these studies have generally relied on limited sample sizes or phenotypic evaluations, constraining a comprehensive understanding of genome-wide diversity patterns. In contrast, high-throughput genotyping using DArTseq single nucleotide polymorphism (SNP) markers has been successfully applied to characterize genetic diversity and population structure in other legume crops, including cowpea [26], chickpea [27], and common bean germplasm from Ethiopia [28]. These studies demonstrate the power of DArTseq technology to resolve genetic relationships and identify valuable diversity for crop improvement, yet its application to common bean landraces from the Peruvian Amazon remains largely unexplored. The Peruvian Amazon harbors a wide array of common bean landraces and wild relatives, which are essential for preserving genetic diversity. The adaptation of common bean to the tropical rainforest conditions is particularly important due to the challenges associated with high humidity, variable soil fertility, and significant pathogen pressure. However, no systematic or representative collection has been established from the Peruvian Amazon, nor has a comprehensive analysis of the genetic diversity across its rainforest regions been conducted. As a result, little is known about the genetic diversity and origins of these cultivars, which may represent an untapped reservoir of alleles for crop improvement. This study aimed to assess the genetic diversity of common bean landraces collected in the Peruvian Amazon using both morphological descriptors and genome-wide SNP markers (DArTseq). By combining phenotypic and molecular approaches, we sought to clarify the structure of local germplasm and to generate information that supports both conservation strategies and future breeding efforts.

Materials and Methods

Sample Collection

A total of 1,025 common bean (*P. vulgaris*) accessions were recently collected from 10 rainforest regions of Peru: Amazonas, Cajamarca, San Martín, Huánuco, La Libertad, Loreto, Ucayali, Cerro de Pasco, Junín, and Cusco across the Peruvian Amazon, including Indigenous farming systems and traditional local markets (Fig. 1). From this collection, a subset of 633 accessions—representative from Amazonian region—was selected for morphological and molecular characterization. Also, it was added 34 accessions for genotyping as a reference genetic material belonging to both Mesoamerican and Andean gene pools, looking for the relationships between accessions and to help

to define gene pool group; from them 26 are from Peru (12 from Coast, 14 from Highlands), 7 from CIAT (2 Andean y 5 Mesoamerican gene pool), and 1 from Mexico which was considered as a reference for Mesoamerican gene pool.

The map was elaborated using R program, according the following methodological approach for creating the map of bean accessions in Software R consisted of the following sequence: 1) obtaining elevation data (DEM) for the Peruvian territory using the get_elev_raster() function from the elevatr package; 2) loading political boundary vector layers via rnaturalearth; 3) importing and converting collected bean occurrence points—stored in clipboard data—into a spatial format (sf); and 4) integrating all layers into a two dimensional map with ggplot2, overlaying the shaded relief (elevation raster with a customized color palette), departmental borders, collection locations and cartographic elements such as a north arrow and scale bar. Thereafter, the map was exported as a TIF image.

Figure 1. Distribution of Common bean accession from Peruvian Amazonia.

Morphological Characterization

The experimentation was conducted in 2024 cropping seasons at the experimental field of Universidad Nacional Agraria La Molina (UNALM), Lima, Peru. The density of sowing was in a 2.5 m row: 35 cm between plants and 50 cm between row, with up to three seeds per hole (in total by experimental unit, 7 holes and 21 plants). Phenotypic data were recorded for each accession following the standardized common bean (Phaseolus vulgaris L.) descriptor guidelines established by the International Board for Plant Genetic Resources [29]. Traits evaluated included plant growth habit, and pod characteristics (shape, color, size, and presence of sutures or fiber). Throughout the growing cycle, a total of 18 traits were recorded—3 quantitative and 15 qualitative—covering vegetative, floral, pod, and seed characteristics (Table 1); such as seed shape, size, color, and plant growth habit were evaluated according to standard descriptors. For each accession, five plants were evaluated, and from each plant, 10 pods and ten seeds were analyzed. Pods were collected at full physiological maturity. The evaluated descriptors and their corresponding measurement criteria were as follows:

Growth habit: Determined based on plant architectural characteristics, using the descriptive categories and reference diagrams provided in the descriptor manual.

Days to flowering: Recorded as the number of days from sowing to the opening of the first floral bud in at least 50% of the evaluated plants per accession.

Standard color: Assessed on freshly opened flowers collected from the inflorescence at the fourth node of each evaluated plant, using the standardized descriptor color chart.

Wing color: Recorded from freshly opened flowers collected from the inflorescence at the fourth node of each evaluated plant, following the descriptor color chart.

Green pod color: Evaluated on ten fully developed immature pods per accession, using the descriptor color chart.

Pod length: Calculated as the mean pod length (cm) based on measurements of ten pods collected from five representative plants per accession.

Pod cross-section: Determined by examining a transverse section of ten fully developed immature pods at the level of the second seed from the distal end, following the descriptor reference chart.

Pod curvature: Assessed on ten fully developed immature pods per accession, according to the descriptor description chart.

Number of locules: Calculated as the mean number of locules determined from the longest pod of each of ten pods collected from five plants per accession.

Pod wall fiber: Evaluated on ten pods collected from five plants per accession, using the descriptor model to classify fiber development.

Pod apex position: Recorded from ten pods collected from five plants per accession, following the descriptor model.

Pod apex orientation: Assessed on the same set of ten pods per accession, based on the descriptor model.

Pod color at physiological maturity: Evaluated on ten dry pods at harvest, using the standardized descriptor color chart.

Seed coat pattern: Recorded from ten seeds per accession, each taken from the seed closest to the pod apex, using the descriptor pattern chart.

Seed coat lighter color: The primary (lighter) seed coat color was recorded from ten seeds per accession, each taken from the seed closest to the pod apex, using the descriptor color chart.

Seed coat darker color: The secondary (darker or background) seed coat color was recorded from the same ten seeds per accession, following the descriptor color chart.

Seed brightness: Assessed as seed coat luster on ten seeds per accession, each taken from the seed closest to the pod apex.

Seed shape: Recorded from ten seeds taken from the middle portion of each pod, following the descriptor classification scheme.

Morphological data analysis

Prior to statistical analyses, quantitative variables were summarized by calculating arithmetic means, while qualitative variables were summarized using their modal values. The resulting data matrix was subsequently used for both univariate and multivariate analyses. Quantitative traits were described using boxplots and summary statistics, including mean, minimum, and maximum

values, to characterize the range and distribution of phenotypic variation. Qualitative traits were coded according to standardized descriptor scales and graphically represented using frequency histograms to visualize their distribution across accessions. A principal component analysis (PCA) was used to examine the association between the analyzed traits and the similarity among accessions, and Multiple Correspondence Analysis (MCA) was made in order to associate qualitative variables to the groups. PCA was performed with all quantitative traits and qualitative categorized values. Additionally, for all traits, a Gower's distance was computed for clustering analysis by using the Ward.D2 method. All statistical analyses and graphical visualizations were performed using R version 4.5.1 [30] and RStudio version 2025.05.1+513 [31], programming language for statistical computing and data visualization, using R Base and packages as "FactoMineR" [32], "factoextra" [33], "ggplot2" [34], and "randomForest" [35].

Table 1. Quantitative and qualitative traits recorded in the *Phaseolus vulgaris* accessions.

Adapted from IBPGR—International Board for Plant Genetic Resources

Genotyping

DNA isolation and sequencing

Seeds of each common bean accessions were germinated in a shade house. Leaf samples were collected from two-week-old seedlings, immediately frozen at −80 °C, and used for DNA extraction. Genomic DNA was extracted from frozen leaf tissue using CTAB protocol. DNA quality and concentration were initially assessed using a NanoDrop spectrophotometer and subsequently quantified with a Qubit fluorometer (Life Technologies, Carlsbad, CA, USA). DNA Integrity was verified via 0.8% agarose gel electrophoresis. The DNA concentration was adjusted to 50–100 ηg µl−1 and samples were submitted for genotyping using high-throughput genotyping DArTSeq™ technology platform at SAGA-CIMMYT(Mexico), following the protocol described [36] and [37]. This technology uses a complexity reduction approach based on a digestion/ligation reaction, employing a combination of two restriction enzymes, PstI and MsI, to enrich genomic representations with single-copy sequences. PstI-compatible adapters were ligated for subsequent PCR amplification, along with sample-specific barcodes added to the digested fragments. Equimolar amounts of amplified products from each sample were pooled and sequenced on an Illumina Novaseq 6000 platform. Sequence data were processed using proprietary DArT analytical pipelines to filter low-quality reads and assign sequences to individual samples based on barcode information.

SNP calling and genotypic data filtering

Molecular markers discovery was performed using an analytical pipeline (DArTsoft14) developed by DArT company, this software identifies two types of markers: SilicoDArT (presence/absence) and SNP markers [38]. Marker scoring and reproducibility were assessed using technical replicates, and only markers with high call rates and reproducibility were retained. Both markers were aligned to the Phaseolus vulgaris YP4 reference genome [1] to identify the chromosome positions. Initially, we received 80,079 DArTSeq-derived SNP markers from SAGA-CIMMYT, which were polymorphic across common bean genotypes analyzed. Markers with unknown position were first removed from

the analysis. Marker parameters such as call rate and minimum allele frequency (MAF) were calculated using dartR package in R version 4.5.1 [39]. Accordingly, SNPs markers with ≥ 50% call rate and MAF of ≥ 0.5% were retained for further analysis. In this study, genotypes with missing data above ≥ 30% were removed from the analysis. For SilicoDArT markers (presence / absence), the initial report of 462,250 markers was filtered to retain markers with call rate ≥ 90% and MAF of ≥ 0.5%. Markers with unknown position were also removed from the final analysis.

Genetic relationships and diversity metrics

Pairwise genetic distances among accessions were calculated using the scaled Euclidean distance method, as implemented in the gl.dist.ind function of the dartR package. Genetic diversity indices, including expected heterozygosity (He), observed heterozygosity, polymorphic information content (PIC), major allele frequency, and inbreeding coefficient (Fis), were computed using functions also available in dartR package.

To infer relationships among accessions, we constructed a neighbor-joining tree, based on the previous euclidean distance matrix, in DARwin v6.2.1. [40]. Principal Coordinate Analysis (PCoA) was conducted using the gl.pcoa function in dartR to visualize genetic relationships among accessions in reduced dimensions.

Population structure and genetic diversity analysis

Population structure was inferred using fastSTRUCTURE, a framework for inferring population structure from a large SNP genotype dataset [41]. Analyses were conducted for a range of K values (number of assumed populations) from 1 to 10. The ancestry proportions for each individual (Q-matrix) were visualized alongside the neighbor-joining tree and combined with the geographical passport data to contextualize cluster assignments.

Results and discussion

Nominal variability of common bean

Based on passport data collected during fieldwork a total of 74 different common names were recorded for common bean from Peruvian Amazonia. The ten most frequently cited cultivars were Panamito, Waska, Ashpa, Pinto, Canario, Poroto, Ñuña (popping bean), Shingo, Manteca and Awisho (Table 2). Of these, Panamito and Canario are commercial cultivars likely introduced from the coastal region. These are predominantly cultivated in higher-altitude zones by migrant farmers from the coast and highlands. The remaining cultivars are traditional landraces distributed across both the lowland and upland Amazon. The most common variety collected was *Panamito*, representing 15.4% of all accessions. This was followed by local landraces such as *Waska* (12.4%), *Ashpa* (11.8%), and *Pinto* (6.5%). The four bean cultivars most widely cultivated in the Peruvian Amazon collectively accounted for only 5.4% of the total cultivated material. In contrast, 17 cultivars (representing 23% of the total) exhibited low frequencies (1–4%), whereas the majority—70 cultivars—were cultivated at frequencies below 1%, together comprising 71.6% of the recorded landraces. This highly skewed frequency distribution indicates that most Amazonian bean landraces are maintained under localized and low-intensity cultivation systems. Such a pattern underscores their vulnerability to genetic erosion and highlights the need to explicitly incorporate rarity and spatial restriction into strategies for the conservation, management, and effective utilization of Amazonian common bean germplasm.

Table 2. Nominal variability of common bean from Peruvian Amazonia.

Morphological variability

We describe the characterization of 476 accessions (from 667 sowed), the rest of 191 accessions did not progress in the field trial. Based on qualitative and quantitative morphological descriptors, we estimated the variability of the collected accessions. Substantial phenotypic variability was observed among the accessions, particularly in traits such as seed color and growth habit, reflecting both natural selection and farmer-driven selection (Fig. 2).

Figure. 2. Morphological variability of common bean from Peruvian Amazonia, showing different form, size, color of seed and its ripe pod.

Boxplot for quantitative traits shows high variability. Flowering time ranged from 32 to 75 days, showing that some common bean landraces have short vegetative growth periods, which would be important for use in the development of early-maturing cultivars. In the case of pod length, the range was from 6.5 to 17.3 cm and locules per pod ranged from 2 to 11, which are very important source for common bean plant breeders, offering important yield components to develop improved cultivars for the tropical rainforest (Fig. 3).

Figure 3. Boxplot of quantitative descriptors on 476 common bean accessions

a= Days to flowering, b = Pod length, and c= Number of locules per pod

The histogram analysis of qualitative traits revealed substantial phenotypic variation in flower, pod, and seed characteristics, including color, size, and shape (Fig. 4). All growth habit categories defined in the descriptor list were represented. Indeterminate bush types were the most frequent, followed by determinate bush types, whereas indeterminate prostrate and indeterminate climbing forms occurred at low frequencies. This distribution suggests a predominance of plant architectures commonly associated with shorter vegetative growth cycles in environments such as the Peruvian Amazon.

Flower color traits showed clear patterns of variation. White was the predominant standard color, followed by green, lilac, white with lilac margins, and purple. Wing color exhibited a similar distribution, with white being the most common, followed by green, lilac, and purple.

Pod color was mainly green, with additional variation including purple-striped green, carmine-striped green, and pale red–striped green pods. Pod cross-section was predominantly pear-shaped, followed by round-elliptic, very flat, and Fig-eight forms. Slightly curved pods were most frequent, followed by straight and curved pods.

Regarding pod wall fibers, excessive shattering—characterized by strong twisting of dry pods—was the most common condition, followed by leathery pods, in which dry pods do not open spontaneously. The pod apex position was mainly marginal, and apex orientation was predominantly downward (ventral).

At physiological maturity, pod color was primarily light yellow to white, followed by golden or deep yellow, pale yellow with purple mottling or stripes, deep yellow with purple mottling, and pale yellow with red mottling.

Considerable diversity was observed in seed traits. All seed coat pattern states were detected, with whole color being the most frequent, followed by rhomboid-spotted, striped, speckled, broad-striped, and bicolor patterns. Variation was also evident in seed coat lighter color, predominantly yellow to greenish, followed by black, carmine red or wine, pure white, pale cream to buff, and gray. Seed coat darker color was mainly carmine red or wine and black, with additional classes including yellow to greenish, brownish to greenish, pale cream to buff, purple, and pink.

Seed brightness was predominantly intermediate, followed by matte and shiny types. Seed shape was mainly cuboid, followed by truncate-fastigate, oval, round, and kidney-shaped forms.

Overall, the high level of qualitative trait diversity observed in this collection highlights its value as a genetic resource for the conservation, utilization, and improvement of common bean adapted to Amazonian environments.

Figure 4. Histogram of 15 qualitative descriptors on 476 common bean accessions.

Relationships and variability

Cluster analysis revealed the presence of four well-defined groups among common bean (*Phaseolus vulgaris* L.) accessions from the Peruvian Amazon (Fig. 5). This clear partitioning indicates a structured pattern of morphological diversity within these landraces. Cluster formation was driven by 18 discriminant descriptors—three quantitative and 15 qualitative—that captured substantial variation in plant architecture, phenology, and seed-related traits. The observed diversity reflects not only underlying genetic differentiation but also the influence of farmer-mediated selection and adaptation to contrasting agroecological conditions. The main characteristics defining each group are summarized below.

Group 1: Short vegetative cycle and small Seed

Accessions in this group are characterized by early flowering and rapid physiological maturity, coupled with the lowest 100-seed weight among all clusters. Plants predominantly exhibit a determinate growth habit, reduced vegetative development, and relatively small leaflets, traits typically associated with early-cycle and low-input production systems.

Group 2: Large seed and climbing habit

This group comprises accessions with the largest seed size and highest seed weight. An indeterminate climbing growth habit (Type IV) predominates, characterized by long internodes and pronounced vining capacity. These accessions tend to exhibit later maturity, extended vegetative growth, and a higher number of pods per plant, reflecting their suitability for longer growing cycles and supported cropping systems.

Group 3: High yield potential and vegetative vigor

Accessions are distinguished by superior performance in key yield-related traits, including seeds per pod and total grain weight per plant. Plants display vigorous vegetative growth, dense canopy development, broad leaflets, and stems with high mechanical strength. Growth habits are mainly Type II or III, suggesting a favorable balance between vegetative biomass and reproductive allocation.

Group 4: Intermediate Morphological Types

This group includes accessions with intermediate values for quantitative descriptors. Differentiation within this cluster is primarily driven by qualitative traits, such as flower color and seed-coat patterning. These accessions appear to represent transitional or recombinant morphotypes, potentially acting as a genetic bridge between more extreme phenotypic classes.

The identified clusters were not strictly associated with geographic origin; however, certain accessions, such as Awisho and Ashpa, consistently grouped together and were predominantly cultivated in lowland environments. This pattern suggests incipient morphological differentiation linked to ecological factors, particularly altitude-related environmental gradients. Overall, the structured diversity observed among these landraces highlights their considerable potential for use in breeding programs aimed at improving yield and adaptation, while simultaneously conserving the rich agrobiodiversity of the Peruvian Amazon.

Figure 5. Tree of 476 common bean accessions according to 18 morphological descriptors

The color indicates the groups: group 1 = green, group 2 = orange, group 3 = grey, and group 4 = pink.

The first two principal components (PC1 and PC2) explained 16.9% and 11.2% of the total phenotypic variation, respectively, accounting for a cumulative 28.1% (Fig. 6). The relatively low proportion of variance captured by the first two components indicates a high level of morphological complexity among the evaluated accessions, with the remaining 71.9% of variation distributed across subsequent components. This pattern suggests that multiple traits with moderate contributions collectively shape phenotypic differentiation, rather than a few dominant variables.

The PCA biplot revealed the presence of four groups, broadly consistent with the clusters identified through hierarchical cluster analysis. However, clear separation based on morphological traits was evident only for groups 1 and 4. Group 1 was characterized by late flowering (approximately 50 days after sowing), excessive shattering of pod wall fibers, kidney-shaped and truncate–fastigiate seed forms, and a relatively high number of locules per pod (approximately 8). In contrast, group 4 comprised early-flowering accessions (approximately 38 days), strongly contracting pod wall fibers, predominantly round to oval seed shapes, and a lower number of locules per pod (approximately 5.6).

In contrast, groups 2 and 3 showed substantial overlap in the multivariate space, with no clear morphological boundaries between them. This lack of separation suggests phenotypic admixture or shared trait combinations among these groups, potentially reflecting gene flow, parallel selection

by farmers, or convergent adaptation to similar agroecological conditions. Overall, the PCA results support the cluster analysis while highlighting that only a subset of morphological traits contributes to clear phenotypic differentiation among the identified groups.

Figure 6. The two first PC biplot of 476 common bean accessions according to 18 morphological descriptors.

The color indicates the groups: group 1 = green, group 2 = orange, group 3 = grey, and group 4 = pink.

Multiple Correspondence Analysis (MCA), performed using the SNP-defined subgroups, showed that, in general, the subgroups were not distinguished by exclusive qualitative descriptors (Fig. 7). This result indicates a high degree of overlap in qualitative morphological traits among genetically defined groups. An exception was observed for subgroup 1.2, which was associated with lilac flower coloration (standard and wings), light red pod color at physiological maturity, and a carmine seed coat color. This subgroup corresponds to the Andean gene pool, suggesting that, in this case, certain qualitative descriptors are partially congruent with the genetic structure revealed by SNP data.

Figure 7. Multiple Correspondence Analysis (MCA) of 476 accessions with 15 qualitative descriptors.

The color indicates the groups and sub-groups according to the SNP-defined subgroups.

SNP marker variation and distribution

From the initial set of 665 common bean accessions, 647 presented less than 30% missing data and were retained for downstream analyses. SNP markers were filtered to retain only those with a call rate of at least 50% and a minor allele frequency (MAF) of 0.5% or higher, yielding a filtered dataset of 24,283 high-quality SNPs (Fig. 8, Table 3). Alignment to the common bean YP4 reference genome revealed that 23,050 SNPs (94.9%) were successfully mapped to chromosome positions.

For SilicoDArT markers, the applied filter of Call rate ≥ 90% and MAF of ≥ 0.5% produced a set of 29,865 markers. Additionally, alignment to YP4 genome reference revealed that 22,594 SilicoDArT markers (75.7%) were successfully mapped to chromosome positions. Results of statistical analysis were very similar than obtained with SNP markers, which is shown as supplementary material (S1-S4 Figs).

Figure 8. Histogram distribution of single nucleotide polymorphism (SNP) marker parameters

A) Callrate, B) Frequency of heterozygous genotypes, C) Read depth, D) Polymorphic Index Content (PIC), and E) Minimum allele frequency (MAF) on the filtered set of 23050 SNP markers.

Table 3. Single nucleotide polymorphism (SNP) call rate report pre and post filtering

Genetic relationships and Population Structure

Clustering analysis based on the neighbor-joining algorithm resolved the evaluated accessions into two well-defined major groups, reflecting their underlying genetic relationships (Fig. 9A). The first group comprised primarily accessions collected from the Peruvian Amazon, together with reference accessions included as an outgroup of Andean origin from the Peruvian highlands, the coastal region, and CIAT. The second group consisted mainly of Peruvian Amazon accessions, along with outgroup materials of Mesoamerican origin, including CIAT accessions and a reference sample from Mexico. This clear bipartite structure indicates that the first group corresponds to the Andean gene pool, whereas the second group represents the Mesoamerican gene pool, consistent with the recognized primary domestication centers of common bean.

Principal Coordinate Analysis (PCoA) further supported this genetic differentiation, with the first three axes explaining 62.62%, 8.97%, and 2.77% of the total molecular variation, respectively (Fig. 9B). The first axis clearly separated the Andean and Mesoamerican gene pools, while the second and third axes captured additional within-group variation. These results confirm the strong genetic structure within the collection and highlight the coexistence of both major gene pools among common bean landraces cultivated in the Peruvian Amazon.

Population structure analysis provided additional evidence for this pattern. The Evanno method identified the strongest signal of genetic structure at K = 2 (Fig. 9D), in agreement with the cluster analysis and PCoA results. These two genetic clusters correspond to the Andean and Mesoamerican gene pools and likely reflect contrasting domestication histories, evolutionary trajectories, and adaptation processes associated with their geographic origins. At a higher level of resolution (K = 4), two subgroups were detected within each major group (Fig. 9B), revealing finer-scale genetic differentiation. Notably, these subgroups showed a strong association with farmers' common names for the cultivars, providing valuable insight into the historical diffusion, local adaptation, and cropping practices of common bean in the Amazonian region (Fig. 10).

Within the Andean gene pool (Group 1), two subgroups were identified and designated as subgroup 1.1 and subgroup 1.2. Subgroup 1.1 included recently collected accessions together with reference materials from the Andean highlands (including popping beans or nuñas), and coastal regions, which were incorporated to elucidate genetic relationships among Peruvian bean germplasm. This subgroup predominantly comprised accessions known locally as Canario, Norteño, Manteca, Pinto, Chuncho, 60 Días, and Caballero. In contrast, subgroup 1.2 consisted exclusively of recently collected accessions from the Peruvian Amazon and was mainly represented by landraces referred to as Ashpa, Panamito, Ashpilla, and Vaca Paleta (Fig. 10). The genetic distinctiveness of this subgroup suggests local differentiation and adaptation under Amazonian agroecological conditions.

Similarly, the Mesoamerican gene pool (Group 2) was subdivided into subgroup 2.1 and subgroup 2.2. Subgroup 2.1 comprised exclusively recently collected Amazonian accessions, predominantly cultivated under the common names Waska, Awisho, and Poroto. Subgroup 2.2 included both Amazonian accessions and outgroup materials of Mesoamerican origin and was mainly composed of landraces known as Panamito, Shingo, Cambio 90, Pozuzo, Pinto, and Garrapata (Fig. 10). The presence of both local and reference materials within this subgroup indicates historical introductions and subsequent integration of Mesoamerican germplasm into Amazonian farming systems.

Overall, the combined clustering, PCoA, and population structure analyses reveal a complex genetic architecture of common bean landraces in the Peruvian Amazon, shaped by the coexistence of the Andean and Mesoamerican gene pools, localized diversification, and farmer-mediated selection. These findings underscore the importance of Amazonian landraces as reservoirs of genetic diversity and highlight their potential value for conservation and breeding programs targeting adaptation to diverse and changing environments.

Figure 9. Genetic structure of 647 common bean accessions with SNPs 23,050 markers.

A) Neighbor joining tree showing two main clusters, ancestry proportions from STRUCTURE analysis from K2 to K5 (left to right). B) Principal Coordinate Analysis colored according to the two main clusters of the NJ tree. C) Pie chart of the ancestry proportions of K=4 for each accession with geographic coordinates. D) Evanno plot showing the strongest signal of population structure at K=2.

Figure 10. Number of common names distribution in each subgroup of common bean from Amazonia, Peru.

Genetic Diversity (α and β) using "q" profiles

Using the two main groups identified in the Peruvian Amazon common bean panel, we quantified within-group (alpha, α) and between-group (beta, β) diversity across Hill numbers for q = 0, 1, and 2 based on the 23,050 SNP markers. The results are shown in the Table 4.

For q = 0 (allelic richness), α was 1.97 in Group1 and 1.95 in Group2, which is expected in biallelic SNPs, while β was 1.04, indicating that both groups harbor essentially the same set of allelic states with virtually no private alleles unique to either group. At q = 1 (Shannon diversity), α was identical in both groups (1.31) and β increased to 1.20, which indicates that although within-group allele-frequency balance is similar, the groups begin to differ in how common those alleles are between populations. At q = 2 (Gini–Simpson/expected heterozygosity), α was 1.22 in Group1 and 1.23 in Group2, whereas β rose to 1.4–1.5, indicating a clearer differentiation driven primarily by shifts in the frequencies of common alleles.

Taken together, these q-profiles show minimal presence/absence differentiation but progressively stronger frequency-based divergence from q = 0 to q = 2, indicating that the genetic structuring between groups is explained more by differences in the prevalence of shared alleles than by unique allelic repertoires.

Table 4. Hill number of α (intrapopulation) and β (interpopulation) diversity indices according to the 23,050 loci SNP and two groups of common beans from Amazonia, Peru.

Using the same 23,050 SNPs, we extended the q-profile analysis to four populations defined by previous cluster and STRUCTURE analysis. The results of the analysis in the four subgroups are shown in Table 5.

For q = 0, α ranged narrowly from 1.82 to 1.94 (see Table 5), indicating that all populations harbor nearly the full set of biallelic states with only minor differences in allelic richness. At q = 1 , α values differentiated the populations more clearly (Subgroup1_1 = 1.31; Subgroup1_2 = 1.25; Subgroup2_1 = 1.16; Subgroup2_2 = 1.30), which indicates varying degrees of allele frequency balance: Subgroup1_1 and Subgroup2_2 exhibit more even allele frequencies, whereas Subgroup2_1 shows greater skew toward common alleles. At q = 2, the ranking persisted (Subgroup2_1 = 1.10; Subgroup1_2 = 1.16; Subgroup2_2 = 1.21; Subgroup1_1 = 1.22), indicating that differences among populations are driven primarily by the frequencies of common alleles.

Taken together, the four-way comparison shows minimal differences in allelic repertoires (q = 0) but increasing divergence at q = 1 and q = 2, which indicates that genetic differentiation among these subpopulations is explained less by presence/absence of allelic states and more by shifts in the prevalence and balance of shared alleles.

Comparing the two subgroups within each main group we observe little presence/absence differentiation but a clear frequency-based divergence. For subgroup1_1 versus subgroup1_2, β increased from 1.06 at q = 0 to 1.24 at q = 1 and ~1.29 at q = 2, which indicates that while both subgroups share essentially the same allelic states, they differ modestly to moderately in allele-frequency balance, with the strongest contrast driven by common alleles. A similar behavior is observed for subgroup2_1 versus subgroup2_2 (Table 5).

Table 5. This is the Table 5 Title. Hill number of α (intrapopulation) and β (interpopulation) diversity indices according to the 23,050 loci SNP and four subgroups of common beans from Amazonia, Peru.

Population Heterozygosity

The parameters of diversity using heterozygosity, according to the 23,050 loci SNP, two-groups and four subgroups of common beans are shown in the Table 6.

Two-groups analysis

Group 1: where the effective sample size was ~240 individuals, showing 22,281 polymorphic loci (almost all, 97% of the total). The Ho = 0.041, meaning that on average, ~4.1% of the loci per individual are heterozygous. Then, the He/uHe = ~0.148, under Hardy-Weinberg Equilibrium (HWE) shows around ~14.8% of heterozygous loci would be expected. The FIS = 0.52 (inbreeding coefficient), indicated a severe heterozygote deficit. The population is less heterozygous than expected, which reflect inbreeding, that is a expected result in a self-pollinating populations as a common bean.

Group 2: The effective sample size was ~318 individuals, showing 21,846 polymorphic loci (94.8% of the total). The Ho = 0.035, around ~3.5% of loci are heterozygous on average, even lower than group 1. The proportion He/uHe= ~0.147 is similar to group 1 in expected heterozygosity; and FIS= 0.54, also shows strong heterozygote deficit, very comparable to group 1. Comparing both Group 1 vs. Group 2, they show very similar He (~0.15), indicating that the allele pool and frequencies are similar. Ho is lower in group 2 than group 1(0.035 vs. 0.041), suggesting that individuals in group 2 express less heterozygosity. The FIS is elevated in both populations (>0.5), reinforcing the idea of a generalized heterozygote deficit, which is expected for common bean populations. Finally, group2 has more monomorphic loci than group 1 (1,204 vs 769), which could reflect loss of variation or fixation at certain sites.

Four subgroups analysis

The subgroup1_1 has effective size ~136 individuals and 21,511 polymorphic loci (93% of the total). The Ho = 0.032, quite low compared to He = 0.145; and the FIS = 0.58, reflecting a strong heterozygote deficit.

The subgroup 1_2 has effective size ~104 individuals and 20,444 polymorphic loci (88.7%), more monomorphic loci than subgroup1_1. The Ho = 0.053, higher than subgroup1_1, individuals show slightly more heterozygosity. However, the He = 0.115 was lower than subgroup1_1; and the FIS = 0.35, still positive but lower heterozygote deficit than in subgroup1_1.

Subgroup2_1 has effective size ~131 individuals and 18,877 polymorphic loci (82%), the lowest of the four subgroups. Also, the Ho = 0.029 and He = 0.071 were the lowest of all subgroups, indicating a loss of diversity; and the FIS = 0.30 showed a moderate heterozygote deficiency.

Subgroup2_2 has effective size ~187 individuals and 21,563 polymorphic loci (93.5%), comparable to subgroup1_1. The Ho = 0.040 showed an intermediate value between subgroup1_1 and subgroup1_2; and the He = 0.139 was intermediate too; and FIS = 0.53, indicating a severe heterozygote deficiency.

Comparisons between subgroups within the group.

Group_1: the subgroup1_1 has a higher He (0.145) but a lower Ho, resulting in a very high FIS (0.58). While, the subgroup1_2 has a lower He (0.115) but a relatively higher Ho (0.053), which reduce the deficit of heterozygosity (FIS = 0.35). The subgroup1_1 appears more structured as an self-pollinating population, while subgroup1_2 maintains more heterozygotes despite lower overall diversity.

Group 2: the subgroup2_1 has the lowest diversity among the four subgroups (Ho = 0.029; He = 0.071). However, the subgroup2_2 recovers diversity (He = 0.139), but its FIS is still high (0.53). Within Group_2, there is a clear inequality; subgroup2_1 present loss of diversity; and subgroup2_2 is more diverse but lacks heterozygotes.

In all four subgroups, there is a clear disconnection between Ho and He, all subgroups have fewer heterozygotes than expected under Hardy–Weinberg equilibrium. Also, the positive and high FIS (0.30–0.58) in all subgroups suggest inbreeding, internal structure favoring homozygotes, which is expected in common bean.

Table 6. This is the Table 6 Title. Genetic diversity parameters within groups and subgroups with the 23,050 loci SNP of common beans from Amazonia, Peru.

This is the Table 6 legend. n.Ind (ef.) = effective sample size; n.Loc = number of loci; polyLoc = number of polymorphic loci; monoLoc = monomorphic loci; Ho (Observed heterozygosity; proportion of heterozygous loci per individual, average across the population); He (Expected heterozygosity, Nei 1978; expected heterozygosity under Hardy–Weinberg equilibrium); uHe (unbiased He, corrected for sample size); FIS (Inbreeding coefficient = 1 – Ho / uHe, positive values indicate a deficit of heterozygotes (possible inbreeding or structure) and negative values indicate an excess).

Discussion

Morphologic variability

The morphologic diversity observed in term of type of plant, flowering time, color, size, form of pods and seeds confirms that Amazonian *P. vulgaris* represents an important genetic reservoir. The presence of distinct genetic clusters suggests that both environmental heterogeneity and farmer practices shape phenotypic variability. Similar levels of morphological diversity have been reported in Brazil [42], Mexico [43], Spain [44], Nigeria [45], Pakistan [46], Ethiopia [47]. In Peru, however, most previous studies have focused on highland germplasm, using small sample size [23] [22] [21] [48] and lately sample from Amazonia [25]; which are not representative to allow a comprehensive estimation of common bean variability. Our results, show that much of the morphological diversity is conserved on farms, where farmers save, select, and replant seeds year after year, ensuring both autonomy and household income [49] [50]. In addition, frequent seed acquisition by farmers from traditional markets further promotes gene flow and admixture, particularly relevant under tropical conditions where high temperature and humidity shorten seed longevity, and its storage challenges; causes rapid loss of viability. Together, these practices contribute to the maintenance and renewal of agrobiodiversity in the Peruvian Amazon. It is relevant to highlight the farmer behaviors, who collet new material and sow all together with own local landraces. Then, the agroecological mosaic, coupled with traditional knowledge, has led to the maintenance of a broad range of landraces with varying morphological traits. A large portion of this agrobiodiversity is still conserved *in situ*, within Indigenous and local farming systems. Farmers contribute to the conservation and evolution of these cultivars by using and exchanging seeds year by year [51] [52]. Additionally, field observations and interviews revealed that many farmers regularly acquire seeds from traditional markets to replace planting stock each season. This behavior is likely influenced by tropical environmental constraints—such as high temperatures and humidity—which accelerate seed aging and reduce viability during storage. Thus, constant renewal of planting material becomes a necessary strategy. The morphological diversity recorded—particularly in traits like growth habit, seed color, and pod characteristics—supports the idea of local adaptation and cultural preferences influencing varietal selection. Morphological characterization, though often considered basic, remains a valuable and accessible tool for identifying distinct genotypes and understanding their agronomic potential and farmer preferences.

Molecular diversity

SNP-based analyses, cluster and Principal Coordinate analysis (PcoA) revealed that the accessions were assigned into two distinct groups, corresponding to the Andean or Mesoamerican gene pools,

284 and 363 accessions, respectively (Fig. 9). Of the Amazonia accessions, 56.1 % clustered with Mesoamerican pool and 43.9 % with the Andean gene pools. However, this tendency was largest in others common bean germplasm genetic diversity studies, e.g. in Brazil was reported, more than 70 % of Mesoamerican gene pool, [4]; [53]; and in Ethiopian germplasm, the 284 common bean accessions were roughly classified into Andean and Mesoamerican gene pools, assigning 188 (65.05%) accessions in the Mesoamerican group, while the remaining 96 (33.22%) accessions were classified in the Andean gene pool [28]. The population structure analysis (based on K = 2 groups) showed that the common bean accessions from Peruvian amazon were distributed between two gene pools, Andean and Mesoamerican with a low degree of admixture. By comparison, when considering K = 4, both the Andean and Mesoamerican gene pool was clustered in two subgroups, with low admixture.

This structure is consistent with findings from USDA core collections [54], where PCoA and NJ trees confirmed the Andean–Mesoamerican split and further subdivisions into ecogeographic races. These results indicate that Amazonian germplasm is integrated into the global diversity framework of *P. vulgaris* while retaining unique local substructure. However, in Brazilian germplasm genetic diversity studies in common bean were obtained more diversity in Mesoamerican gene pool using GBS analysis [53]. In overall, the genetic variation detected through DarTseq SNP analysis showed that, the clustering agrees with that obtained by the principal coordinate analysis and population structure analyses.

The high genetic variation detected through DArTseq SNP analysis complements the phenotypic findings and highlights the importance of both *in situ* and *ex situ* conservation. Many of these accessions, particularly those maintained only on-farm, are at risk of being lost due to changes in land use, market pressures, climate change, or generational shifts in farming practices. Comprehensive genetic characterization enables the identification of unique and underrepresented alleles, which can be prioritized for conservation and potentially incorporated into breeding programs aimed at improving resilience to biotic and abiotic stresses.

Alpha (α) and Beta (β) Genetic Diversity

The Peruvian Amazonia common beans, according to the alpha-diversity estimates showed similar internal variability in both gene pools, with most loci being biallelic and rare alleles contributing little or marginal to overall diversity. Beta-diversity revealed that Andean and Mesoamerican groups largely share the same alleles but differ in allele frequencies, reflecting the combined effects of local selection, drift, and demographic histories.

The four subgroups identified at K=4 maintained nearly complete allelic richness, with differentiation arising mainly from differences in allele frequencies rather than unique variants. This pattern is consistent with historical farmer migration from the Andes into Amazonian areas, where likely Andean beans were introduced and later mixed with locally adapted materials, followed by continuous selection and cultivation under tropical conditions. This result is similar what was found by [55]on diversification and population structure in common beans, suggesting geographic isolation, founder effects or natural selection and introgression were involved in creating the diversity of cultivated beans.

Population Heterozygosity

Despite the high proportion of polymorphic *loci*, observed heterozygosity (Ho ≈ 0.035–0.041) was markedly lower than expected (He ≈ 0.147–0.148). Positive and high inbreeding coefficients (FIS = 0.30–0.58) across all groups and subgroups indicate strong homozygosity, which is expected in a predominantly self-pollinating crop such as common bean. Similar patterns of heterozygote deficit have been reported in Brazil and Ethiopia [42] [28].

In both morphological and molecular characterization, no duplicates were found in the material under study, this result is similar that was found evaluating 67 accessions based on morphological traits and molecular markers from Brazil [42]; which suggests the importance of keeping all accessions in the germplasm bank since they represent a valuable source of genetic diversity. Importantly, this variability has remained largely unexploited in breeding programs, which have focused on disease resistance; lagging to obtain high yielding and high-quality cultivars in major production regions [56] [57] Incorporating Amazonian diversity into breeding pipelines could broaden the adaptive base of common bean. Hence, the conservation and systematic use of this germplasm are of strategic importance for Peru national genetic resources and future crop improvement.

Conclusion

This article addresses the under characterization of genetic diversity in common bean (landraces from the Peruvian Amazon, which limits their use in breeding programs. Prior to this study, most research focused on commercial cultivars from highland and coastal regions, neglecting the unique genetic resources found in the Amazon. This study demonstrates that common bean landraces from the Peruvian Amazon harbor remarkable morphological and genetic diversity, confirming the region as a key reservoir of agrobiodiversity. Both morphological and SNP-based analyses consistently revealed two major groups corresponding to the Andean and Mesoamerican gene pools, each subdivided into local subgroups that include unique Amazonian accessions.

There was high allelic richness but low observed heterozygosity, consistent with the self-pollinating nature of the species. Furthermore, genetic diversity analyses showed similar internal variability between groups, with differences arising mainly from allele frequency rather than different allele presence, and confirmed the expected high levels of inbreeding in this predominantly self-pollinated crop. Importantly, no redundant accessions were detected, highlighting the unique value of each collected sample.

The integration of morphological descriptors with DArTseq SNP markers proved effective in identifying structure and patterns of variation, demonstrating the complementarity of these approaches for germplasm management. Because much of this diversity is maintained only *in situ* by local farmers, there is an urgent need to reinforce both *in situ* and *ex situ* conservation strategies.

This study enhances our understanding of the genetic diversity present in Amazonian common beans, highlighting the need for conservation strategies to preserve these valuable resources for future breeding efforts. They provide novel opportunities for breeding programs seeking traits related to tropical adaptation, resilience to climatic stresses, and farmer- and market-preferred characteristics. Harnessing this diversity is essential to ensure food security and sustainable agriculture development in the face of future challenges.

Acknowledgements

The authors are thankful to SAGA- Genetic Analysis Service for Agriculture, CIMMYT-Mexico; for their support for genotyping. Also, we thank local farmers for their cooperation and the research institutions (UNALM, UNSM, UNU) that supported this study.

Author contributions

JT, MB, DV and MP contributed to conceptualization, to methodology and to writing; JP, JC and YV contributed to investigation; DV, RR and CP to data curation, writing—original draft preparation, visualization, and formal analysis; JS contributed to writing—review; JE contributed to review; RO contributed to writing—review and editing; RB contributed to conceptualization, to methodology to supervision and to funding acquisition. All authors have read and agreed to the published version of the manuscript.

Conflicts of interest

The authors declare no conflict of interest.

Ethical approval

This article does not contain any studies with human participants and animals and ethical approval is not applicable.

Data availability

Supplementary Tables 1 and 2 provide the data on morphological characterization and DArTseq single nucleotide polymorphisms. They are available at

Blas, Raul, 2026, "Morphological characterization data of common bean_Amazonia_Peru", https://doi.org/10.17026/LS/6IRCBV, DANS Data Station Life Sciences, DRAFT VERSION

Blas, Raul, 2026, "DArTseq_SNP_647x23050 markers_common_bean_Peru", https://doi.org/10.17026/LS/SWIWOJ, DANS Data Station Life Sciences, DRAFT VERSION

References

1. Wang Y, Xiaopeng H, Chunhai C, Haigang W, Peng G, Xukui Y, et al. Telomere-to-telomere genome of common bean (*Phaseolus vulgaris* L.) YP4. 2025; GigaScience 14: giaf001. Doi: 10.1093/gigascience/giaf001

2. Bitocchi E, Nanni L, Bellucci E, Rossi M, Giardini A, Zeuli PS, et al. Mesoamerican origin of the common bean (*Phaseolus vulgaris* L.) is revealed by sequence data. Agricultural Sciences. 2012; 109: 788–796. Doi: 10.1073/pnas.1108973109

3. Kwak MKJAGP. The putative Mesoamerican domestication center of *Phaseolus vulgaris* is located in the Lerma-Santiago Basin of Mexico. Crop Science. 2009; 49: 554–563.

4. Bellucci E, Bitocchi E, Rau, D, Rodriguez M, et al. Genomics of origin, domestication and evolution of *Phaseolus vulgaris*. In: Tuberosa, R, de, Graner A, Frison E (eds). Springer, Dordrecht, The Netherlands. 2014. Pp. 483–507. Doi: 10.1007/978-94-007-7572-5_20

5. Frascarelli G, Galise T, D'Agostino N, Cafasso D, Cozzolino S, Cortinovis G, et al. The evolutionary history of the common bean (*Phaseolus vulgaris*) revealed by chloroplast and nuclear genomes analysis. Theoretical and Applied Genetics. 2025; 138: 47. Doi: 10.1007/s00122-025-04832-z

6. Singh SP. Broadening the genetic base of common bean cultivars. Crop Science. 2001; 41: 1659–1675. Doi: 10.2135/cropsci2001.1659

7. Gepts P. Origin and evolution of common bean: past events and recent trends. HortScience. 1998; 33: 1124–1130.

8. Schmutz JMP, Wu, GA, Cannon SB, Grimwood J, Jenkins J, Shu S, et al. A reference genome for common bean and genome-wide analysis of dual domestications. Nature Genetics. 2014 ;46: 707–713, 7. Doi: 10.1038/ng.3008

9. Angioi SA, Rau D, Attene G, Nanni L, Bellucci E, Logozzo G, et al. Beans in Europe: origin and structure of the European landraces. Theoretical and Applied Genetics. 2010; 121: 829–843.

10. Paredes M, Becerra V, Tay J. Inorganic nutritional composition of common bean (*Phaseolus vulgaris* L.) genotypes race Chile. Chilean Journal of Agricultural Research. 2009; 69: 486–495.

11. Suárez-Martinez SE, Ferriz-Martínez RA, Campos-Vega R, Elton-Puente JE. Bean seeds: leading nutraceutical source for human health. Journal of Food. 2016; 14: 131–137.

12. Jensen ES, Peoples MB, Boddey RM, Gresshoff PM, Hauggaard-Nielsen H, Alves BJR, et al. Legumes for mitigation of climate change and the provision of feedstock for biofuels and biorefineries. A review. Agronomy for Sustainable Development. 2012; 32: 329–364. Doi: 10.1007/s13593-011-0056-7

13. United Nations. International Year of Pulses, 2016. UN Resolution68/231. 2014. 68th Session General Assembly, New York. https://docs.un.org/en/A/RES/68/231

14. Saburido M, Hererra A. El frijol en la era genómica. Revista Digital Universitaria. 2015; 2: 16.

15. Calero-Hurtado A, Quintero-Rodríguez E, Olivera-Viciedo D, Pérez-Díaz Y, Castro-Lizazo I, Jiménez J, et al. Respuesta de dos cultivares de frijol común a la aplicación foliar de microorganismos eficientes. Cultivos Tropicales. 2018;39: 5-10. Available from: http://scielo.sld.cu/scielo.php?script=sci_arttext&pid=S0258-59362018000300001&lng=es&tlng=pt

16. FAO Legumbres. Pequeñas semillas, grandes soluciones. Food and Agriculture Oganization of the United Nations, Rome, Italy. 2018; 292.

17. Ministerio de Agricultura y Desarrollo Rural, «El agro en cifras. MIDAGRI. 2024. Available: https://siea.midagri.gob.pe/portal/phocadownload/datos_estadisticas/mensual/Agro/2024/Agro_en_cifras%2006%202024.pdf

18. Genesys. Genesys Global Portal on Plant Genetic Resources. 2021. Available: www.genesys-prg.org.

19. I.N.I.A. Catálogo de ñuña en el banco de germoplasma del INIA. 2021. Available: https://hdl.handle.net/20.500.12955/2112

20. I.N.I.A. Catálogo de frijol en regiones Andinas del banco de germoplasma del INIA. 2023. Available: https://hdl.handle.net/20.500.12955/1404

21. Balarezo JC. Evaluación Agromorfológica Y Caracterización Molecular de la ñuña (*Phaseolus vulgaris* L. Idesia (Arica). 2009;27: 29-40. doi: 10.4067/S0718-34292009000100005

22. Marmolejo K. Variabilidad genética del frijol común tipo ñuña (*Phaseolus vulgaris* L.) en las localidades de Carhuaz y Chiquián, Ancash. M.Sc. Thesis. Universidad Nacional Agraria La Molina. 2018. Available: http://repositorio.lamolina.edu.pe/handle/UNALM/3398

23. Amaringo S. Caracterización fenotípica de 5 ecotipos de frijol común (*Phaseolus vulgaris* L.) en el fundo Aucaloma – Lamas. BSc. Thesis. Universidad Nacional de San Martín. Tarapoto; 2015. Available: http://hdl.handle.net/11458/635

24. Collado LA. Diversidad cultivada y socio-cultural en la Amazonia central del Perú. M.Sc. Thesis. Universidad Nacional Agraria de la Selva. 2002. Available: http://repositorio.unas.edu.pe/handle/UNAS/900

25. Vásquez J, Vilca-Valqui NC, Malqui R, Fernández E, Duarez E, Ayala R. Agromorphological characterization of *Phaseolus* spp., accessions in Amazonas region, Peru. Bioagro. 2024;36: 129–142. Doi: 10.51372/bioagro362.1

26. Seo E, Kim K, Jun T, Choi J, Kim S, Sun H. Population structure and genetic diversity in Korean cowpea germplasm based on SNP markers. Plants. 2020; 9: 1190. Doi: 10.3390/plants9091190

27. Verma S, Gupta S, Bandhiwal N, Kumar T, Bharadwaj C, Bhatia S. High-density linkage map construction and mapping of seed trait QTLs in chickpea (*Cicer arietinum* L.) using genotyping-by- sequencing (GBS). Scientific Reports. 2015 ;5: 17512. Doi: 10.1038/srep17512

28. Gelaw YM, Eleblu JSY, Ofori K, Fenta BA, Mukankusi C, Emam EA, et al. High-density DArTSeq

SNP markers revealed wide genetic diversity and structured population in common bean (*Phaseolus vulgaris* L.) germplasm in Ethiopia. Molecular Biology Reporter. 2023;50: 6739-6751. doi: 10.1007/s11033-023-08498-y

29. International Board for Plant Genetic Resources (IBPGR), *Phaseolus vulgaris* descriptors. International Board for Plant Genetic Resources, Rome, Italy. 1982:32.

30. Team RC. R: a language and environment for statistical computing. Vienna, Austria: R Foundation for Statistical Computing; 2025. Available: https://www.R-project.org/

31. Posit team, RStudio: Integrated Development Environment for R. PBC, Boston, MA: Posit Software; 2025. Available: http://www.posit.co/

32. Le S, Josse J, Husson F. FactoMineR: An R package for multivariate analysis. Journal of Statistical Software. 2008; 25: 1-18. Doi: 10.18637/jss.v025.i01

33. Kassambara A, Mundt F. Factoextra: extract and visualize the results of multivariate data analyses. 2020. Doi: 10.32614/CRAN.package.factoextra

34. Wickham H. ggplot2: Elegant graphics for data analysis. Springer-Verlag New York; 2016.

35. Liaw A, Wiener M. Classification and Regression by randomForest. R News. 2002;2: 18–22. Available: https://CRAN.R-project.org/doc/Rnews/

36. Sansaloni C, Petroli C, Jaccoud D, Carling J, Detering F, Grattapaglia D, et al. Diversity Arrays Technology (DArT) and next-generation sequencing combined: genome-wide, high throughput, highly informative genotyping for molecular breeding of Eucalyptus. BMC Proceedings. 2011;5(P54). doi: 10.1186/1753-6561-5-S7-P54

37. Kilian A, Wenzl P, Huttner E, Carling J, Xia L, Blois H., et al. Diversity Arrays Technology: A generic genome profiling technology on open platforms. In: Pompanon F, Bonin A, editors. Data Production and Analysis in Population Genomics. Doi: 10.1007/978-1-61779-870-2_5

38. Petroli C, Kilian A. Introduction to the DArTseq genotyping method and its data outputs. CIMMYT Research Data & Software Repository Network. 2019; v1. Available: https://hdl.handle.net/11529/10548358/

39. Gruber B, Unmack PJ, Berry OF, Georges A. dartr: An Rpackage to facilitate analysis of SNP data generated from reduced representation genome sequencing. Molecular ecology resources. 2018; 18: 691–699.

40. Perrier X, Jacquemoud-Collet JP. DARwin software: Dissimilarity analysis and representation for Windows. 2006. Available: https://darwin.cirad.fr/

41. Raj A, Stephens M, Pritchard JK. fastSTRUCTURE: variational inference of population structure in large SNP data sets. Genetics. 2014;197: 573–589.

42. Paula E, Almeida RN, O. Santos T, Souza Neto JD, Riva-Souza EM, Posse SCP, et al. Genetic Diversity of common bean (*Phaseolus vulgaris* L.) landraces based on morphological traits and

molecular markers, Plants. 2024;13: 2584. Doi: 10.3390/plants13182584

43. García-Narváez AL, Hernández-Delgado S, Hernández-Delgado JL, Mayek-Pérez N, Morphological and agronomic variability of bean germplasm cultivated in Oaxaca, México. Revista bio ciencias. 2020; 7.

44. Arteaga S, Yabor L, Torres J, Solbes E, Muñoz E, Díez, MJ. Morphological and Agronomic Characterization of Spanish Landraces of *Phaseolus vulgaris* L. Agriculture. 2019; 9(7):149.

45. Kouam E, Kamga-Fotso A-A, Anoumaa M. Exploring agro-morphological profiles of *Phaseolus vulgaris* germplasm shows manifest diversity and opportunities for genetic improvement. Journal of Agriculture and Food Research. 2023;14. Doi: 10.1016/j.jafr.2023.100772

46. Nawaz I, Zeb T, Zeb SB, Sherani, J. Evaluation of common beans (*Phaseolus vulgaris* L.) land races based on qualitative traits derived from Himalayan, Andean and Mesoamerican regions. Sarhad Journal of Agriculture. 2022; 38(1):275–286.

47. Tigist SG, Sibiya J, Amelework A, Keneni G. Agromorphological and physiological performance of ethiopian common bean (*Phaseolus vulgaris* L.) genotypes under different agroecological conditions. Plants. 2023;12(12): 2342. https://doi.org/10.3390/plants12122342

48. Castro R, Quispe D. Caracterización morfológica de genotipos de frijol (*Phaseolus vulgaris* L.) en San Juan de Uchubamba-Jauja, La Merced Perú. BSc. Thesis. Universidad Nacional Daniel Alcides Carrión. 2017. Repositorio Universidad Nacional Daniel Alcides Carrión (UNDAC).

49. Figueiredo Menezes Cavalcanti, T., Pombo Sudré, C., Wesley da Silva Corrêa, J., dos Santos Bento, C., Knoblauch Viega de Andrade, E., Kazue Nakamura Fukuji, K., Rodrigues, R. Custodians of common bean diversity in Rio de Janeiro state, Brazil: revealing their socioeconomic and environmental profile. Agroecology and Sustainable Food Systems. 2021; 45: 1165–1188. Doi: 10.1080/21683565.2021.1888186

50. de Avila J, Coelho F, Sardo P, Haddade I, Moonen A. In situ conservation of agrobiodiversity carried out by traditional family farmers in a mountainous region of Brazil. Agroecology and Sustainable Food Systems. 2024; 49(6):797–820, Doi: 10.1080/21683565.2024.2445729

51. Castañeda-Álvarez NP, Haan S, Juárez H, Khoury CK, Achicanoy HA, Sosa CC, et al. Ex situ conservation priorities for the wild relatives of potato (*Solanum* L. Section Petota). PLoS ONE. 2015; 10: 0122599. Doi: 10.1371/journal.pone.0122599

52. Bellon MR Planting hybrids, keeping landraces: agricultural modernization and tradition among small-scale maize farmers in Chiapas, Mexico. World Development. 2011; 39(8):1434–1443. Doi: 10.1016/j.worlddev.2010.12.010.

53. Elias J, Gonçalves-Vidigal M, Ariani A, Valentini G, Martiniano-Souza M, Bisneta MV, et al. Genome-environment association analysis for bio-climatic variables in common bean (*Phaseolus vulgaris* L.) from Brazil. Plants. 2021; 10:1572. Doi: 10.3390/plants10081572

54. Kuzay S. Is the USDA core collection of common bean representative of genetic diversity of the species, as assessed by SNP diversity. *Crop Science*. 2020;60: 1398–1414. Doi:

10.1002/csc2.20032

55. Blair MW, Soler A, Cortés, AJ. Diversification and population structure in common beans (*Phaseolus vulgaris* L.). PLoS ONE. 2012; 7(11): e49488. doi: 10.1371/journal.pone.0049488

56. Singh SP, Schwartz HF, «Breeding common bean for resistance to diseases: a review. Crop Science. 2010; 50: 2199–2223. Doi: 10.2135/cropsci2009.03.0163.

57. Cichy KA, Porch TG, Beaver JS, Cregan P, Fourie D, Glahn, RP, et al. A Phaseolus vulgaris Diversity Panel for Andean Bean Improvement. Crop Science. 2015; 55:2149–2160. Doi: 10.2135/cropsci2014.09.0653

Supporting information

Figure S1 Histogram distribution of SilicoDArT marker parameters

A) Callrate, B) OneRatio, C) Polymorphic Index Content (PIC), and E) minimum allele frequency (MAF) on the initial filtered ser 29865 SNP markers.

Figure S2. Dendrogram of 647 accessions of common bean, according to the 29865 SilicoDArT markers.

Figure S3. Principal coordinate analysis (PCoA) of 647 accessions of common bean according to SilicoDArT markers.

Figure S4. Population structure of 647 accessions of common bean with SNP-DArTseq markers.

K2 to K5 (from left to right)

Figure 1 Click here to access/download;Figure;Fig 1.tif

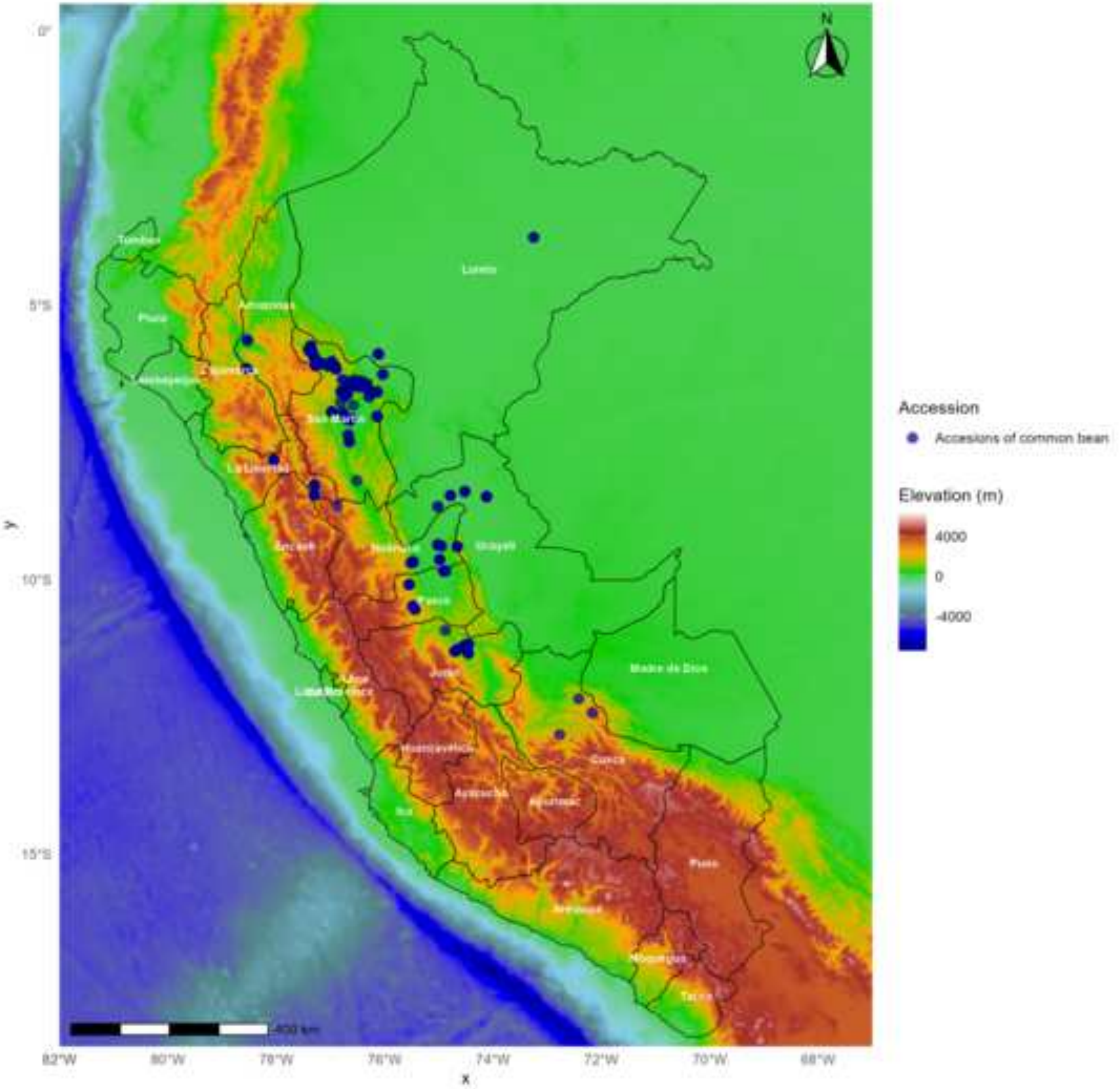

Figure 2

Click here to access/download;Figure;Fig 2.tif

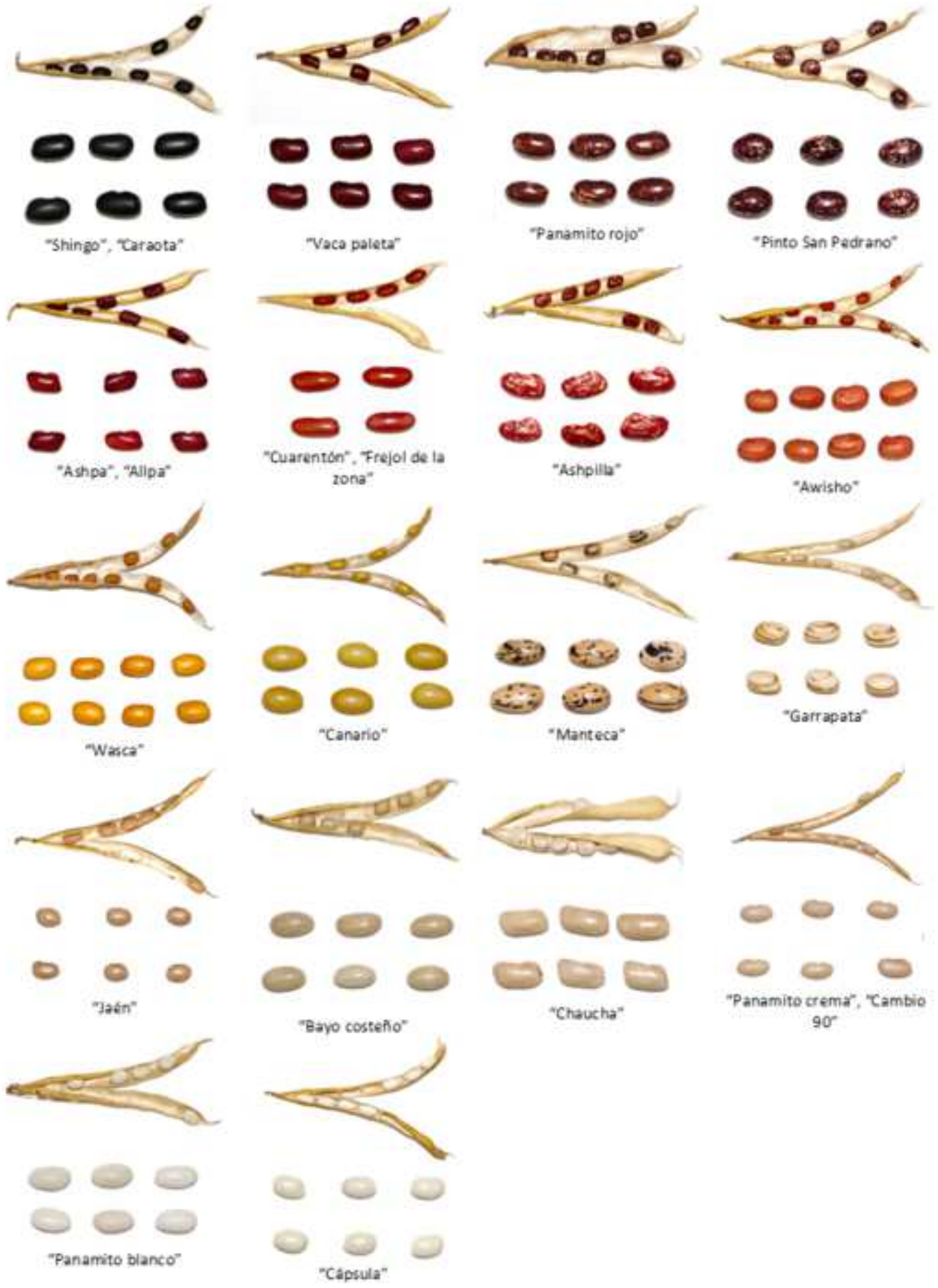

Figure 3

Click here to access/download;Figure;Fig 3.tif

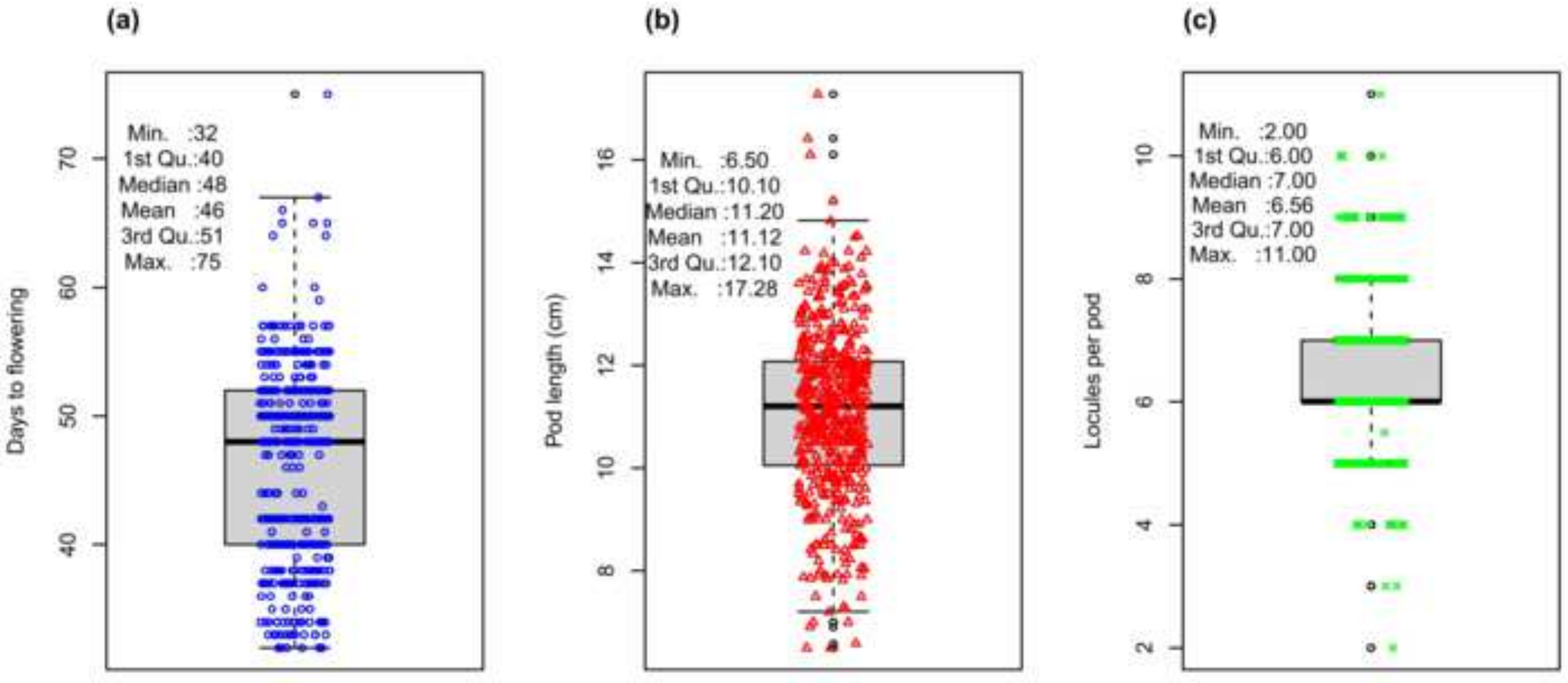

Figure 5

Click here to access/download;Figure;Fig 5.tif

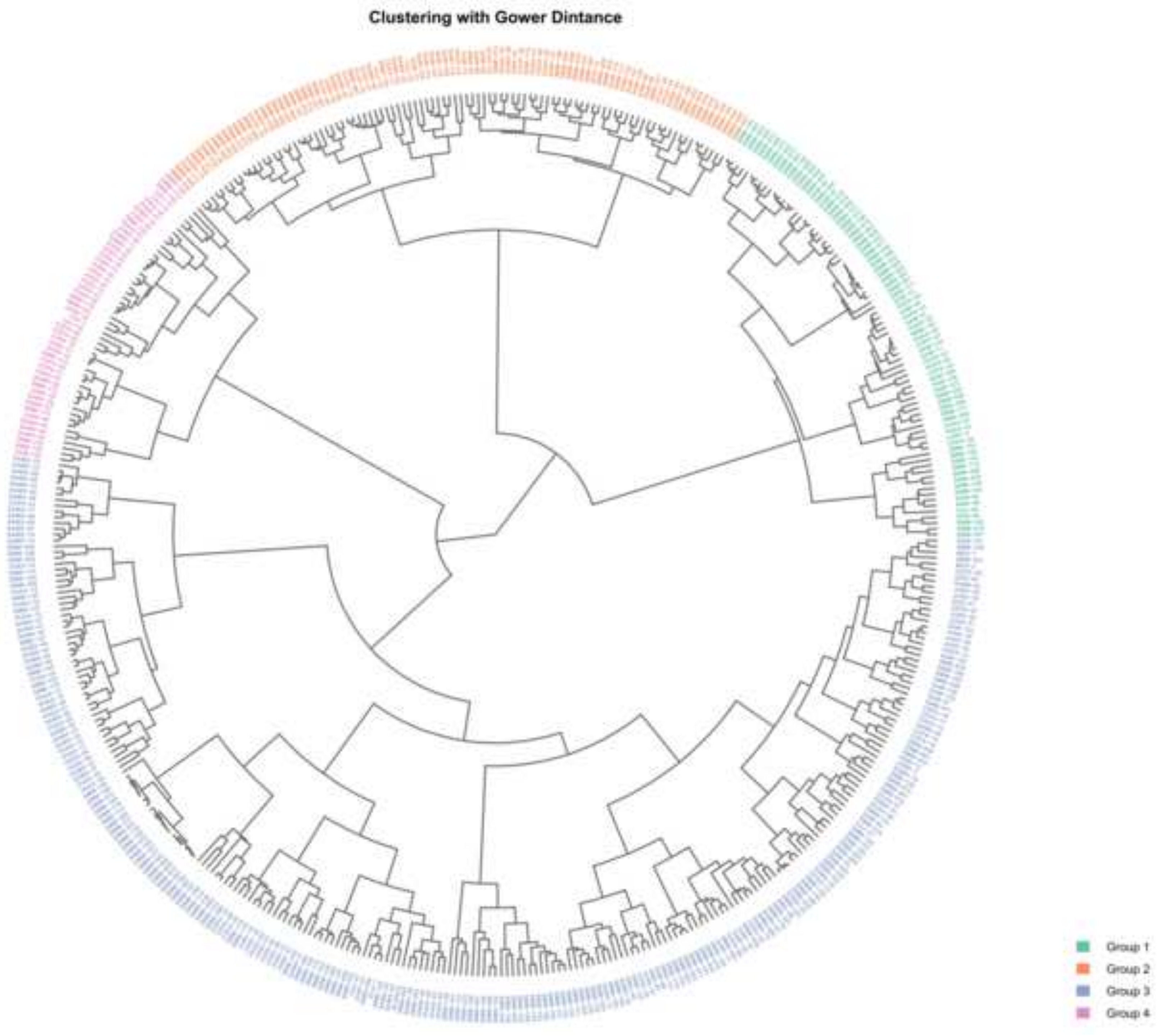

Figure 6

Click here to access/download;Figure;Fig 6.tif

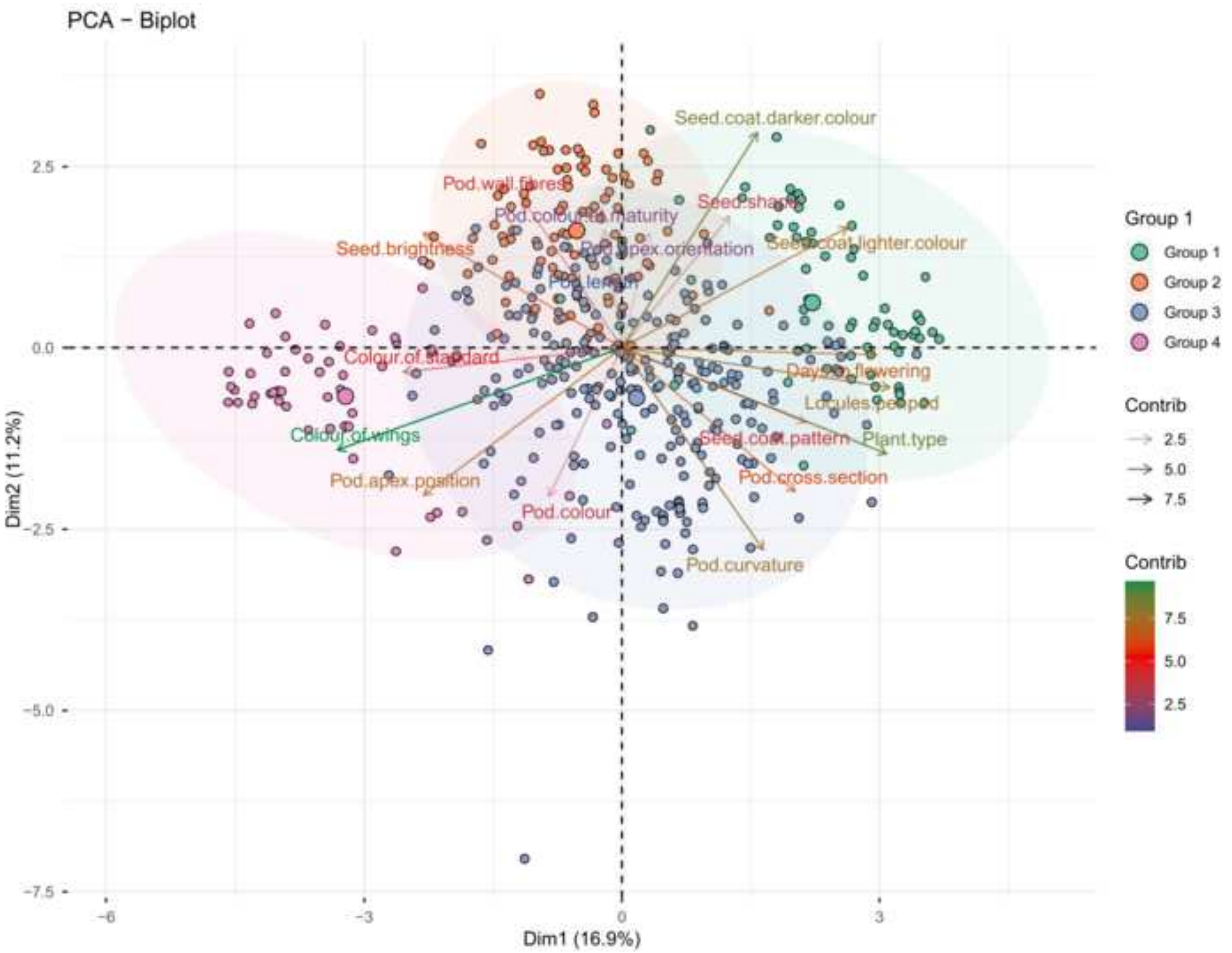

Figure 7

Click here to access/download;Figure;Fig 7.tiff

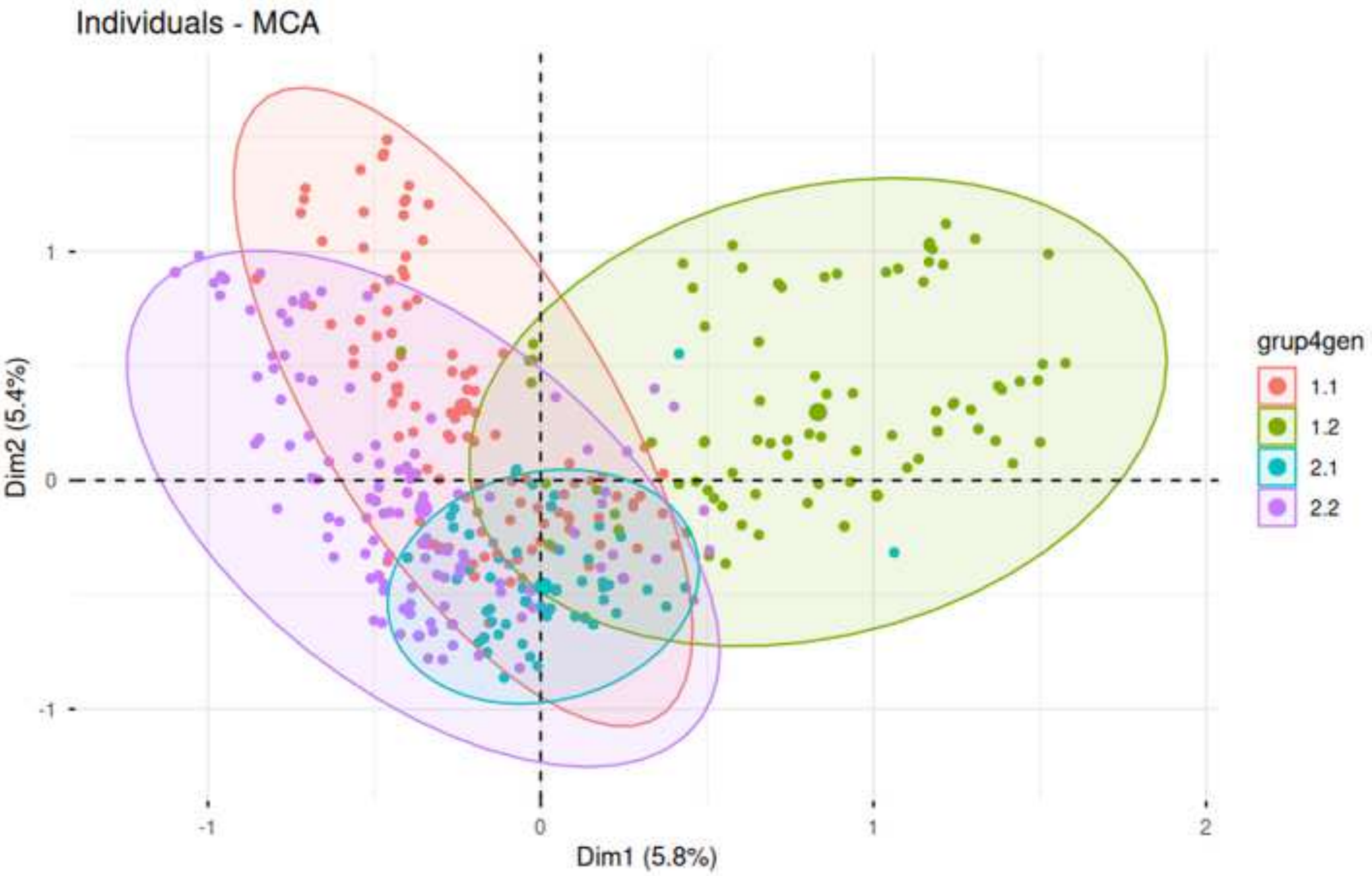

Figure 9

Click here to access/download;Figure;Fig 9.tif

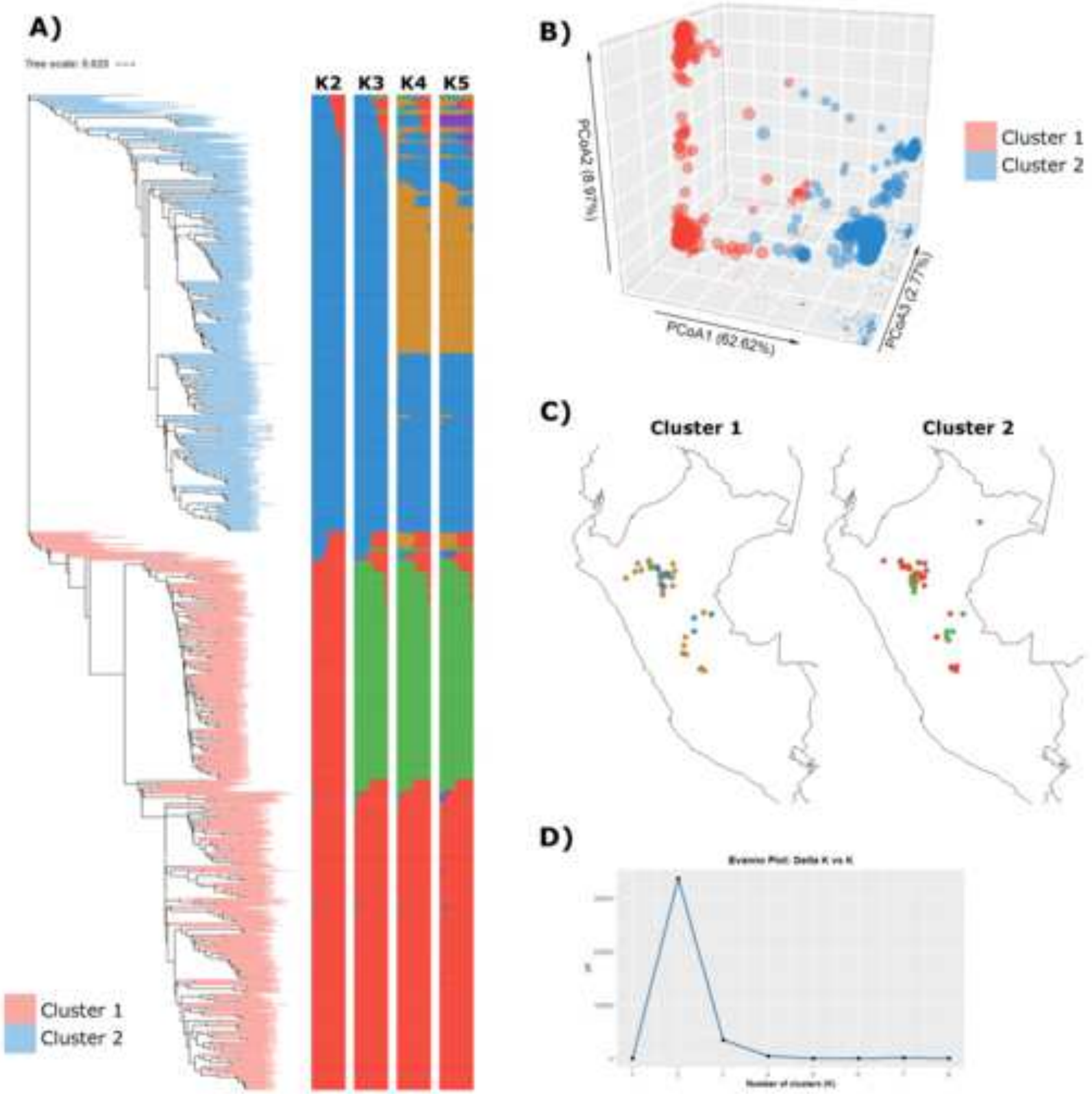

Figure 8

Click here to access/download;Figure;Fig 8.tif

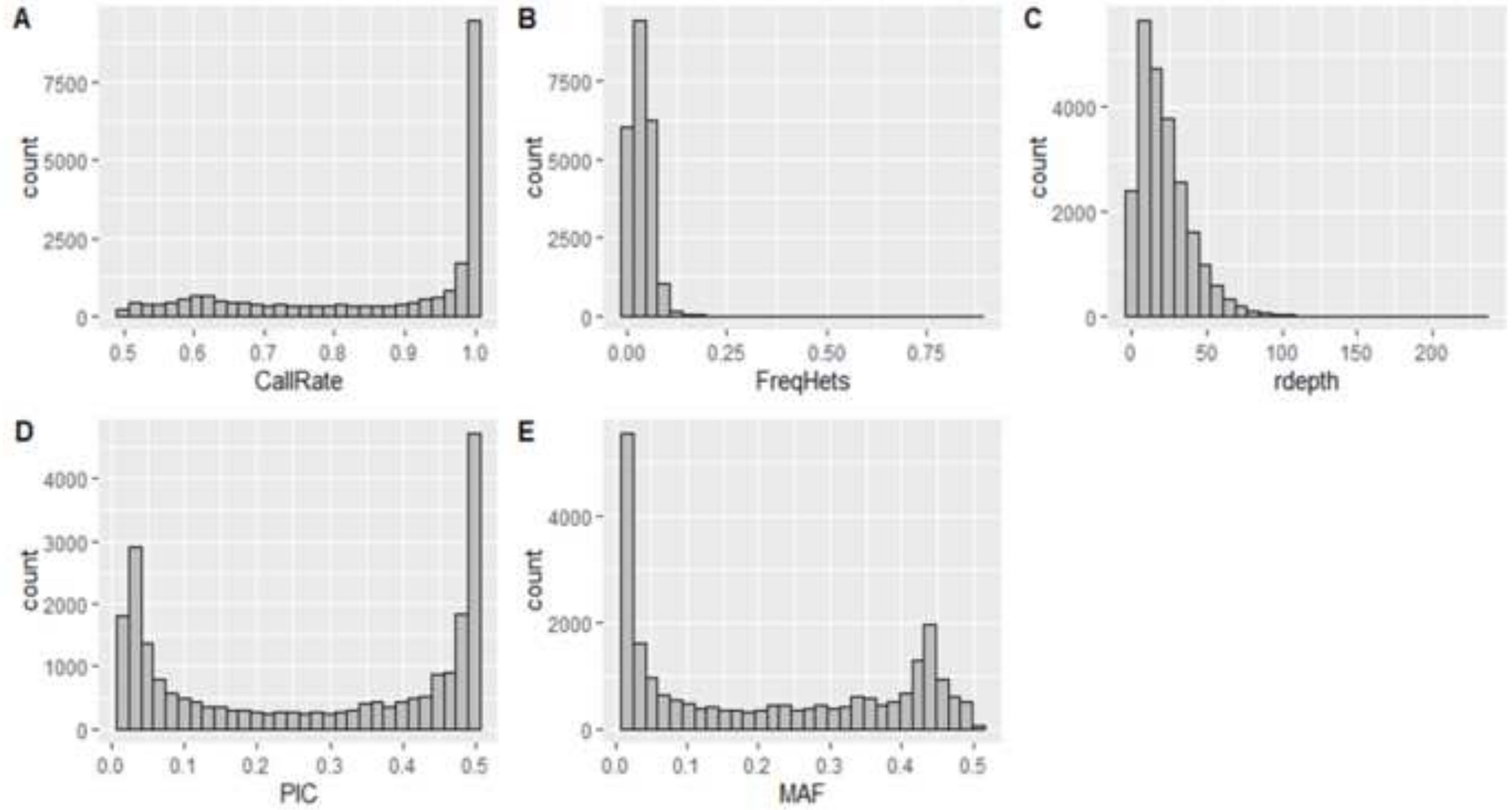

Figure 10

Click here to access/download;Figure;Fig 10.tif

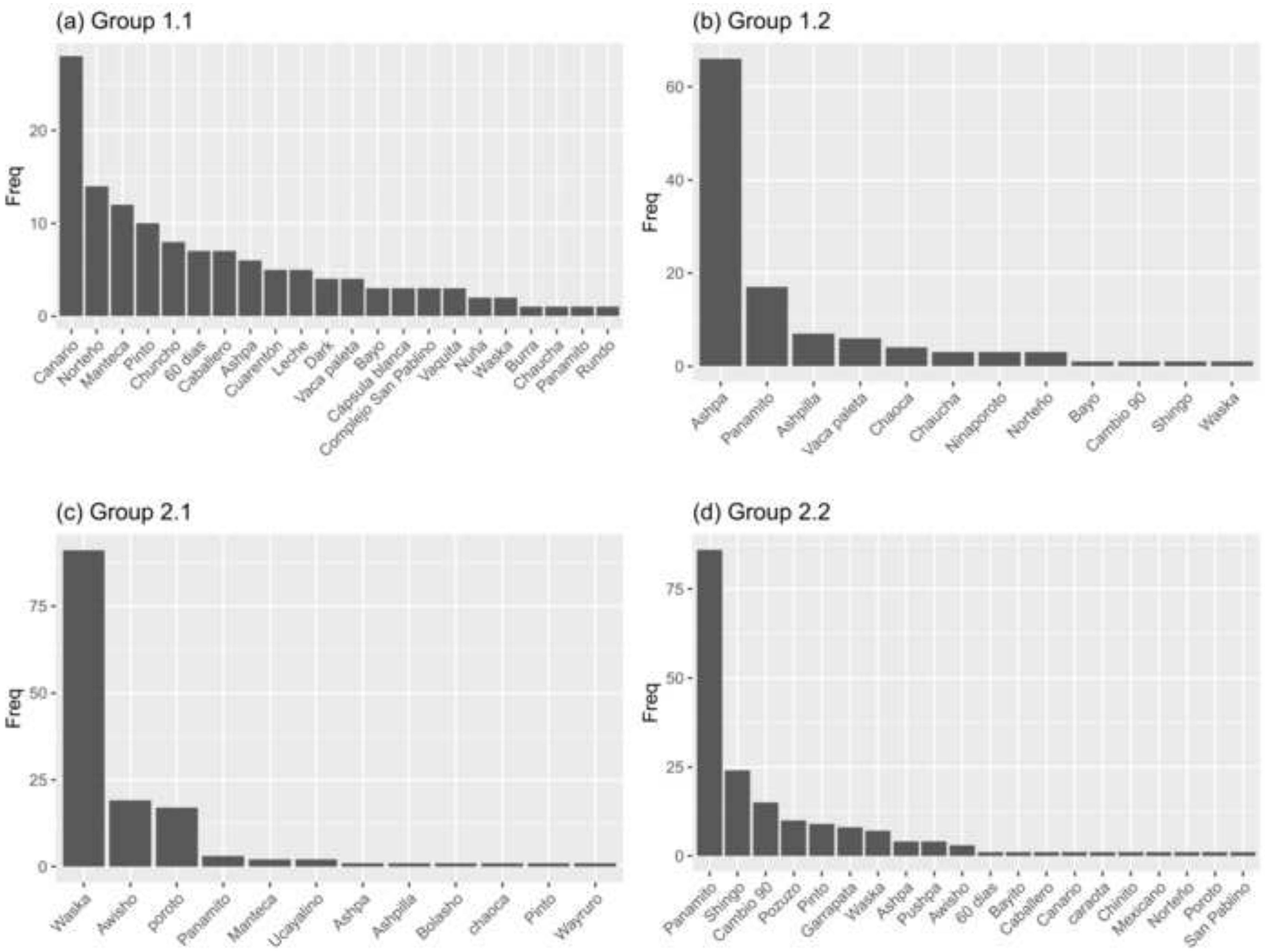

Figure 4

Click here to access/download;Figure;Fig 4.tif

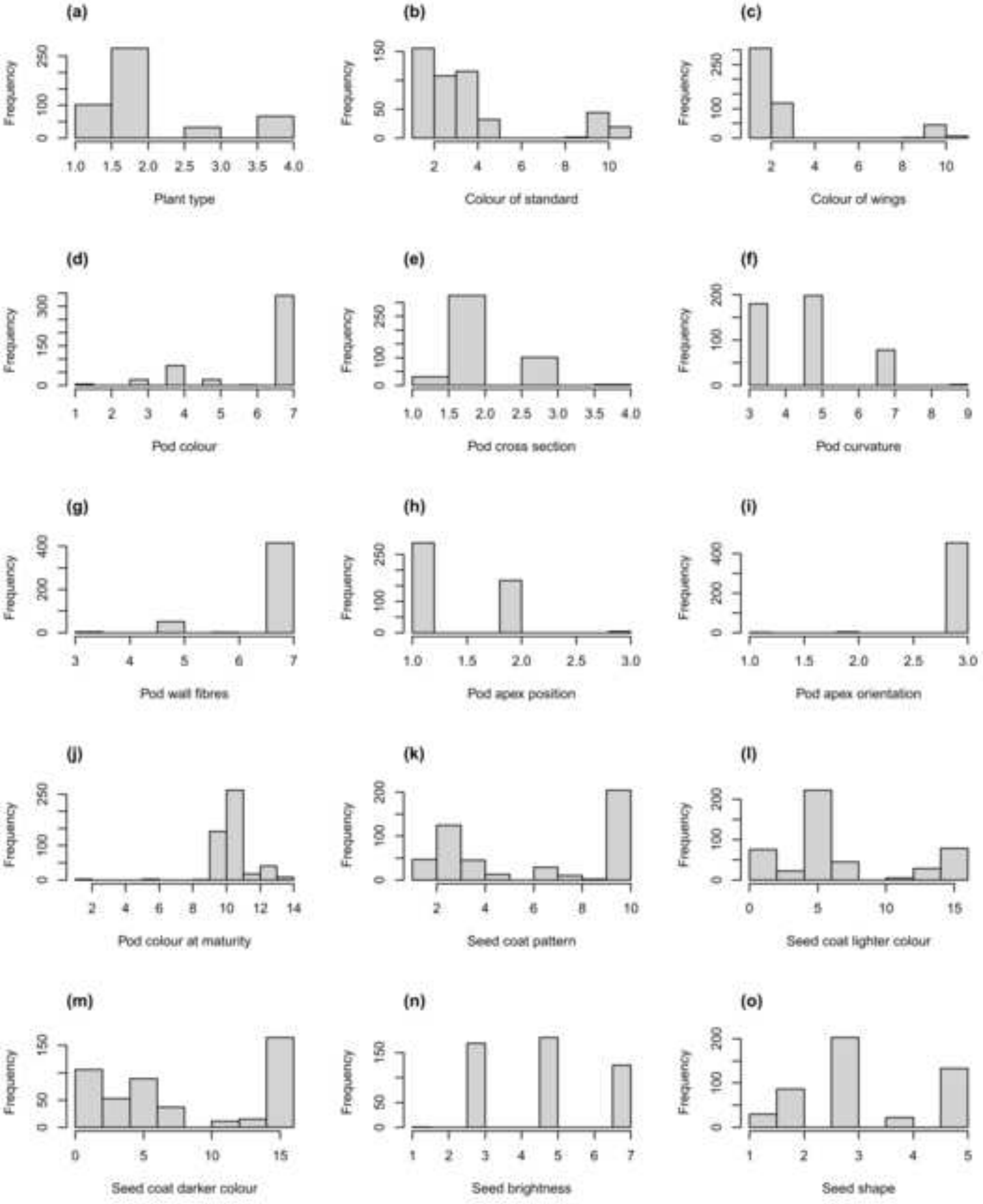

Supporting information Figures S1 to S4

Click here to access/download
Supporting Information
Supporting information-S1.docx

Click here to access/download
Supporting Information
Supplementary_data.docx

Population structure and diversity of common bean (*Phaseolus vulgaris* L.) landraces in the Peruvian Amazon

Jorge Tobaru[1], Marvin Barrera-Lozano[2], Carlos D. Vecco-Giove[2], Jorge Peláez[2], Mack Pinchi[3], Yesenia Vargas[1], Jorge Chuquillanqui[1], Julian Soto-Torres[4], Ronald Robles[4], David Saravia[5], Cesar Petroli[6], Jorge D. Etchevers[7], Rodomiro Ortiz[8] & Raul Blas[1]

[1] Universidad Nacional Agraria La Molina (UNALM), Facultad de Agronomía, Programa de Leguminos de Grano y Oleaginosas. La Molina-Lima, Perú

[2] , Universidad Nacional de San Martín, Facultad de Ciencias Agrarias (UNSM). Tarapoto-San Martín, Perú

[3] Universidad Nacional de Ucayali (UNU), Facultad de Ciencias Agropecuarias. Pucallpa-Ucayali, Perú

[4] International Potato Center (CIP), La Molina, Lima- Peru

[5] Instituto Nacional de Innovación Agraria – INIA, La Molina, Lima- Peru

[6] International Maize and Wheat Improvement Center (CIMMYT), El Batán, Texcoco, Mexico
[7] Colegio de Postgraduados, Texcoco-Mexico

[8] Swedish University of Agricultural Sciences (SLU), Department of Plant Breeding, Alnarp, Sweden

Abstract

Common bean (*Phaseolus vulgaris* L.) is a worldwide important legume with significant nutritional and agronomic value. Despite its global importance, the genetic diversity in Amazonian landraces remains undercharacterized, limiting their utilization in breeding programs. Accordingly, the objective of this study was to comprehensively evaluate the genetic diversity and population structure of common bean populations from the Peruvian Amazon by integrating phenotypic and genomic approaches. A total of 476 accessions were characterized using 18 standardized agro-morphological traits, while a broader set of 647 accessions was analyzed using 23,050 high-quality DArTseq single nucleotide polymorphism (SNP) markers. Our results revealed two major genetic groups corresponding to Andean and Mesoamerican gene pools, each subdivided into distinct subgroups, exhibiting high allelic richness and structured diversity. Despite abundant polymorphic loci, observed heterozygosity was low, consistent with the species' self-pollinating nature. These findings highlight the Peruvian Amazon as a reservoir of unique genetic variation, underscoring the need for integrated in situ and ex situ conservation strategies. This germplasm offers valuable resources for breeding programs targeting tropical adaptation and resilience, contributing to sustainable agriculture and food security.

Keywords: Amazonian landraces, common bean, genetic diversity, morphological characterization, *Phaseolus vulgaris*, plant genetic resources, SNP markers

Introduction

The common bean (*Phaseolus vulgaris* L.) an annual leguminous self-pollinated crop is the most widely cultivated species of the genus Phaseolus and a major source of dietary vegetal protein and income worldwide; which is a diploid (2n = 2x = 22) species and its genome size according to the first telomere-to-telomere (T2T) genome assembly was 560.30 Mb and predicted 29,925 protein-coding genes [1] . The origin of *P. vulgaris* remains controversial, separating it from Mesoamerica and the Andean region. However, recent research using molecular markers, including SNPs, shows that this species' origin is Mesoamerica [2] [3] [4] [5]. Genetic and phylogeographic evidence indicates three distinct wild gene pools: 1) Mesoamerica (Mexico, Central America, Colombia and Venezuela), 2) the Andes (southern Peru, Bolivia and Argentina), and 3) northern Peru/Ecuador; nevertheless, the three common bean genetic groups belong to the primary gene pool [6]. From those, two main domesticated groups emerged belonging to the Mesoamerican and Andean gene pools, this domestication process has been done independently in the Andes and Mesoamerica, which are associated with two Andean and Mesoamerican gen pools, respectively [7] [8]. Both types of beans were introduced to other continents such as Europe, Africa, and Asia, where their widespread acceptance has led to debate about whether Europe can be a secondary center of diversification [9].

At the present, it is a staple crop in many parts of the world, most widely cultivated legume species worldwide, mainly due to its nutritional composition, being rich in amino acids, minerals, and vitamins—especially lysine, iron, zinc and folic acid [10]; presents medicinal attributes such as the prevention of cardiovascular diseases [11]; and has an ecological importance, it fixes atmospheric nitrogen reducing all the environmental costs involved in the acquisition of chemical fertilizers [12]. For this reason, the United Nations declared in 2016 as "the international year of legumes" in its 68th session [13], the common bean being within the legumes, considered one of the essential crops to promote food security. Common bean has thus become an important source of income and nutrients for small farmers in the Caribbean, Latin America, Asia, and Africa, where account for 77% of global production [14] [15] [16]. In the case of Brazil, a predominantly Amazonian region, the Mesoamerican gene pool is found in a greater proportion (79%) and the Andean gene pool in a lesser proportion (21%) [4].

In Peru, the common bean is cultivated across diverse agroecological zones, ranging from coastal valleys to highland and Amazonian regions. According to national statistics [17], production covers approximately 42,575 hectares annually, with San Martín, Piura, and Cajamarca being leading regions; with around 14% of this area located in the Amazon region—2.0% less than the area recorded in 2023. Beyond its economic relevance, the crop plays a crucial role in household nutrition and local food security. Despite its importance, most studies on common bean in Peru have focused on commercial cultivars grown in the highlands and coastal regions, emphasizing agronomic practices and genetic improvement using both local and introduced cultivars. Conversely, there is limited research conducted on the cultivars grown by farmers in indigenous communities of the Peruvian Amazon rainforest. According to the Genesys database, from Peru holds 3,497 accessions of common beans, of which 2,838 (81%) are classified as traditional cultivars, 516 (15%) are results from breeding programs, and 143 (4%) correspond to wild or near-wild forms [18]. The International Center for Tropical Agriculture (CIAT) holds the global reference collection, while the Peruvian National Institute for Agrarian Innovation (INIA) maintains the national bean germplasm bank, which

officially has 58 accessions of beans and 146 accessions of popping bean mainly from the Andean region [19] [20]. Despite these efforts, landraces and wild relatives from the Peruvian Amazon remain underrepresented, leaving a gap in the documentation of local agrobiodiversity.

Research on the diversity of cultivated common bean in Peru remains limited and has largely focused on specific bean types or restricted geographic areas. Most previous studies have emphasized popping beans (ñuña, in the Quechua language), employing phenotypic and molecular markers to characterize their diversity and differentiation [21,22], or have concentrated on isolated localities rather than addressing diversity at a broader regional scale [23]. In the central Peruvian Amazon, cultivated and sociocultural diversity of common bean has been documented, highlighting the role of Indigenous communities and traditional knowledge in shaping bean diversity through local management practices and cultural preferences [24]. More recently, agromorphological characterization of 58 Phaseolus spp. accessions collected in the Amazonas region identified several promising genotypes, with grain yields exceeding 2.77 t ha^{-1}, underscoring their potential value for use in breeding programs [25]. However, these studies have generally relied on limited sample sizes or phenotypic evaluations, constraining a comprehensive understanding of genome-wide diversity patterns. In contrast, high-throughput genotyping using DArTseq single nucleotide polymorphism (SNP) markers has been successfully applied to characterize genetic diversity and population structure in other legume crops, including cowpea [26], chickpea [27], and common bean germplasm from Ethiopia [28]. These studies demonstrate the power of DArTseq technology to resolve genetic relationships and identify valuable diversity for crop improvement, yet its application to common bean landraces from the Peruvian Amazon remains largely unexplored. The Peruvian Amazon harbors a wide array of common bean landraces and wild relatives, which are essential for preserving genetic diversity. The adaptation of common bean to the tropical rainforest conditions is particularly important due to the challenges associated with high humidity, variable soil fertility, and significant pathogen pressure. However, no systematic or representative collection has been established from the Peruvian Amazon, nor has a comprehensive analysis of the genetic diversity across its rainforest regions been conducted. As a result, little is known about the genetic diversity and origins of these cultivars, which may represent an untapped reservoir of alleles for crop improvement. This study aimed to assess the genetic diversity of common bean landraces collected in the Peruvian Amazon using both morphological descriptors and genome-wide SNP markers (DArTseq). By combining phenotypic and molecular approaches, we sought to clarify the structure of local germplasm and to generate information that supports both conservation strategies and future breeding efforts.

Materials and Methods

Sample Collection

A total of 1,025 common bean (*P. vulgaris*) accessions were recently collected from 10 rainforest regions of Peru: Amazonas, Cajamarca, San Martín, Huánuco, La Libertad, Loreto, Ucayali, Cerro de Pasco, Junín, and Cusco across the Peruvian Amazon, including Indigenous farming systems and traditional local markets (Fig. 1). From this collection, a subset of 633 accessions—representative from Amazonian region—was selected for morphological and molecular characterization. Also, it was added 34 accessions for genotyping as a reference genetic material belonging to both Mesoamerican and Andean gene pools, looking for the relationships between accessions and to help

to define gene pool group; from them 26 are from Peru (12 from Coast, 14 from Highlands), 7 from CIAT (2 Andean y 5 Mesoamerican gene pool), and 1 from Mexico which was considered as a reference for Mesoamerican gene pool.

The map was elaborated using R program, according the following methodological approach for creating the map of bean accessions in Software R consisted of the following sequence: 1) obtaining elevation data (DEM) for the Peruvian territory using the get_elev_raster() function from the elevatr package; 2) loading political boundary vector layers via rnaturalearth; 3) importing and converting collected bean occurrence points—stored in clipboard data—into a spatial format (sf); and 4) integrating all layers into a two dimensional map with ggplot2, overlaying the shaded relief (elevation raster with a customized color palette), departmental borders, collection locations and cartographic elements such as a north arrow and scale bar. Thereafter, the map was exported as a TIF image.

Figure 1. Distribution of Common bean accession from Peruvian Amazonia.

Morphological Characterization

The experimentation was conducted in 2024 cropping seasons at the experimental field of Universidad Nacional Agraria La Molina (UNALM), Lima, Peru. The density of sowing was in a 2.5 m row: 35 cm between plants and 50 cm between row, with up to three seeds per hole (in total by experimental unit, 7 holes and 21 plants). Phenotypic data were recorded for each accession following the standardized common bean (Phaseolus vulgaris L.) descriptor guidelines established by the International Board for Plant Genetic Resources [29]. Traits evaluated included plant growth habit, and pod characteristics (shape, color, size, and presence of sutures or fiber). Throughout the growing cycle, a total of 18 traits were recorded—3 quantitative and 15 qualitative—covering vegetative, floral, pod, and seed characteristics (Table 1); such as seed shape, size, color, and plant growth habit were evaluated according to standard descriptors. For each accession, five plants were evaluated, and from each plant, 10 pods and ten seeds were analyzed. Pods were collected at full physiological maturity. The evaluated descriptors and their corresponding measurement criteria were as follows:

Growth habit: Determined based on plant architectural characteristics, using the descriptive categories and reference diagrams provided in the descriptor manual.

Days to flowering: Recorded as the number of days from sowing to the opening of the first floral bud in at least 50% of the evaluated plants per accession.

Standard color: Assessed on freshly opened flowers collected from the inflorescence at the fourth node of each evaluated plant, using the standardized descriptor color chart.

Wing color: Recorded from freshly opened flowers collected from the inflorescence at the fourth node of each evaluated plant, following the descriptor color chart.

Green pod color: Evaluated on ten fully developed immature pods per accession, using the descriptor color chart.

Pod length: Calculated as the mean pod length (cm) based on measurements of ten pods collected from five representative plants per accession.

Pod cross-section: Determined by examining a transverse section of ten fully developed immature pods at the level of the second seed from the distal end, following the descriptor reference chart.

Pod curvature: Assessed on ten fully developed immature pods per accession, according to the descriptor description chart.

Number of locules: Calculated as the mean number of locules determined from the longest pod of each of ten pods collected from five plants per accession.

Pod wall fiber: Evaluated on ten pods collected from five plants per accession, using the descriptor model to classify fiber development.

Pod apex position: Recorded from ten pods collected from five plants per accession, following the descriptor model.

Pod apex orientation: Assessed on the same set of ten pods per accession, based on the descriptor model.

Pod color at physiological maturity: Evaluated on ten dry pods at harvest, using the standardized descriptor color chart.

Seed coat pattern: Recorded from ten seeds per accession, each taken from the seed closest to the pod apex, using the descriptor pattern chart.

Seed coat lighter color: The primary (lighter) seed coat color was recorded from ten seeds per accession, each taken from the seed closest to the pod apex, using the descriptor color chart.

Seed coat darker color: The secondary (darker or background) seed coat color was recorded from the same ten seeds per accession, following the descriptor color chart.

Seed brightness: Assessed as seed coat luster on ten seeds per accession, each taken from the seed closest to the pod apex.

Seed shape: Recorded from ten seeds taken from the middle portion of each pod, following the descriptor classification scheme.

Morphological data analysis

Prior to statistical analyses, quantitative variables were summarized by calculating arithmetic means, while qualitative variables were summarized using their modal values. The resulting data matrix was subsequently used for both univariate and multivariate analyses. Quantitative traits were described using boxplots and summary statistics, including mean, minimum, and maximum

values, to characterize the range and distribution of phenotypic variation. Qualitative traits were coded according to standardized descriptor scales and graphically represented using frequency histograms to visualize their distribution across accessions. A principal component analysis (PCA) was used to examine the association between the analyzed traits and the similarity among accessions, and Multiple Correspondence Analysis (MCA) was made in order to associate qualitative variables to the groups. PCA was performed with all quantitative traits and qualitative categorized values. Additionally, for all traits, a Gower's distance was computed for clustering analysis by using the Ward.D2 method. All statistical analyses and graphical visualizations were performed using R version 4.5.1 [30] and RStudio version 2025.05.1+513 [31], programming language for statistical computing and data visualization, using R Base and packages as "FactoMineR" [32], "factoextra" [33], "ggplot2" [34], and "randomForest" [35].

**Table 1. Quantitative and qualitative traits recorded in the *Phaseolus vulgaris* accessions.**
Adapted from IBPGR—International Board for Plant Genetic Resources

Genotyping

DNA isolation and sequencing

Seeds of each common bean accessions were germinated in a shade house. Leaf samples were collected from two-week-old seedlings, immediately frozen at −80 °C, and used for DNA extraction. Genomic DNA was extracted from frozen leaf tissue using CTAB protocol. DNA quality and concentration were initially assessed using a NanoDrop spectrophotometer and subsequently quantified with a Qubit fluorometer (Life Technologies, Carlsbad, CA, USA). DNA Integrity was verified via 0.8% agarose gel electrophoresis. The DNA concentration was adjusted to 50–100 ηg µl−1 and samples were submitted for genotyping using high-throughput genotyping DArTSeq™ technology platform at SAGA-CIMMYT(Mexico), following the protocol described [36] and [37]. This technology uses a complexity reduction approach based on a digestion/ligation reaction, employing a combination of two restriction enzymes, PstI and MsI, to enrich genomic representations with single-copy sequences. PstI-compatible adapters were ligated for subsequent PCR amplification, along with sample-specific barcodes added to the digested fragments. Equimolar amounts of amplified products from each sample were pooled and sequenced on an Illumina Novaseq 6000 platform. Sequence data were processed using proprietary DArT analytical pipelines to filter low-quality reads and assign sequences to individual samples based on barcode information.

SNP calling and genotypic data filtering

Molecular markers discovery was performed using an analytical pipeline (DArTsoft14) developed by DArT company, this software identifies two types of markers: SilicoDArT (presence/absence) and SNP markers [38]. Marker scoring and reproducibility were assessed using technical replicates, and only markers with high call rates and reproducibility were retained. Both markers were aligned to the Phaseolus vulgaris YP4 reference genome [1] to identify the chromosome positions. Initially, we received 80,079 DArTSeq-derived SNP markers from SAGA-CIMMYT, which were polymorphic across common bean genotypes analyzed. Markers with unknown position were first removed from

the analysis. Marker parameters such as call rate and minimum allele frequency (MAF) were calculated using dartR package in R version 4.5.1 [39]. Accordingly, SNPs markers with ≥ 50% call rate and MAF of ≥ 0.5% were retained for further analysis. In this study, genotypes with missing data above ≥ 30% were removed from the analysis. For SilicoDArT markers (presence / absence), the initial report of 462,250 markers was filtered to retain markers with call rate ≥ 90% and MAF of ≥ 0.5%. Markers with unknown position were also removed from the final analysis.

Genetic relationships and diversity metrics

Pairwise genetic distances among accessions were calculated using the scaled Euclidean distance method, as implemented in the gl.dist.ind function of the dartR package. Genetic diversity indices, including expected heterozygosity (He), observed heterozygosity, polymorphic information content (PIC), major allele frequency, and inbreeding coefficient (Fis), were computed using functions also available in dartR package.

To infer relationships among accessions, we constructed a neighbor-joining tree, based on the previous euclidean distance matrix, in DARwin v6.2.1. [40]. Principal Coordinate Analysis (PCoA) was conducted using the gl.pcoa function in dartR to visualize genetic relationships among accessions in reduced dimensions.

Population structure and genetic diversity analysis

Population structure was inferred using fastSTRUCTURE, a framework for inferring population structure from a large SNP genotype dataset [41]. Analyses were conducted for a range of K values (number of assumed populations) from 1 to 10. The ancestry proportions for each individual (Q-matrix) were visualized alongside the neighbor-joining tree and combined with the geographical passport data to contextualize cluster assignments.

Results and discussion

Nominal variability of common bean

Based on passport data collected during fieldwork a total of 74 different common names were recorded for common bean from Peruvian Amazonia. The ten most frequently cited cultivars were Panamito, Waska, Ashpa, Pinto, Canario, Poroto, Ñuña (popping bean), Shingo, Manteca and Awisho (Table 2). Of these, Panamito and Canario are commercial cultivars likely introduced from the coastal region. These are predominantly cultivated in higher-altitude zones by migrant farmers from the coast and highlands. The remaining cultivars are traditional landraces distributed across both the lowland and upland Amazon. The most common variety collected was *Panamito*, representing 15.4% of all accessions. This was followed by local landraces such as *Waska* (12.4%), *Ashpa* (11.8%), and *Pinto* (6.5%). The four bean cultivars most widely cultivated in the Peruvian Amazon collectively accounted for only 5.4% of the total cultivated material. In contrast, 17 cultivars (representing 23% of the total) exhibited low frequencies (1–4%), whereas the majority—70 cultivars—were cultivated at frequencies below 1%, together comprising 71.6% of the recorded landraces. This highly skewed frequency distribution indicates that most Amazonian bean landraces are maintained under localized and low-intensity cultivation systems. Such a pattern underscores their vulnerability to genetic erosion and highlights the need to explicitly incorporate rarity and spatial restriction into strategies for the conservation, management, and effective utilization of Amazonian common bean germplasm.

Table 2. Nominal variability of common bean from Peruvian Amazonia.

Morphological variability

We describe the characterization of 476 accessions (from 667 sowed), the rest of 191 accessions did not progress in the field trial. Based on qualitative and quantitative morphological descriptors, we estimated the variability of the collected accessions. Substantial phenotypic variability was observed among the accessions, particularly in traits such as seed color and growth habit, reflecting both natural selection and farmer-driven selection (Fig. 2).

Figure. 2. Morphological variability of common bean from Peruvian Amazonia, showing different form, size, color of seed and its ripe pod.

Boxplot for quantitative traits shows high variability. Flowering time ranged from 32 to 75 days, showing that some common bean landraces have short vegetative growth periods, which would be important for use in the development of early-maturing cultivars. In the case of pod length, the range was from 6.5 to 17.3 cm and locules per pod ranged from 2 to 11, which are very important source for common bean plant breeders, offering important yield components to develop improved cultivars for the tropical rainforest (Fig. 3).

Figure 3. Boxplot of quantitative descriptors on 476 common bean accessions

a= Days to flowering, b = Pod length, and c= Number of locules per pod

The histogram analysis of qualitative traits revealed substantial phenotypic variation in flower, pod, and seed characteristics, including color, size, and shape (Fig. 4). All growth habit categories defined in the descriptor list were represented. Indeterminate bush types were the most frequent, followed by determinate bush types, whereas indeterminate prostrate and indeterminate climbing forms occurred at low frequencies. This distribution suggests a predominance of plant architectures commonly associated with shorter vegetative growth cycles in environments such as the Peruvian Amazon.

Flower color traits showed clear patterns of variation. White was the predominant standard color, followed by green, lilac, white with lilac margins, and purple. Wing color exhibited a similar distribution, with white being the most common, followed by green, lilac, and purple.

Pod color was mainly green, with additional variation including purple-striped green, carmine-striped green, and pale red–striped green pods. Pod cross-section was predominantly pear-shaped, followed by round-elliptic, very flat, and Fig-eight forms. Slightly curved pods were most frequent, followed by straight and curved pods.

Regarding pod wall fibers, excessive shattering—characterized by strong twisting of dry pods—was the most common condition, followed by leathery pods, in which dry pods do not open spontaneously. The pod apex position was mainly marginal, and apex orientation was predominantly downward (ventral).

At physiological maturity, pod color was primarily light yellow to white, followed by golden or deep yellow, pale yellow with purple mottling or stripes, deep yellow with purple mottling, and pale yellow with red mottling.

Considerable diversity was observed in seed traits. All seed coat pattern states were detected, with whole color being the most frequent, followed by rhomboid-spotted, striped, speckled, broad-striped, and bicolor patterns. Variation was also evident in seed coat lighter color, predominantly yellow to greenish, followed by black, carmine red or wine, pure white, pale cream to buff, and gray. Seed coat darker color was mainly carmine red or wine and black, with additional classes including yellow to greenish, brownish to greenish, pale cream to buff, purple, and pink.

Seed brightness was predominantly intermediate, followed by matte and shiny types. Seed shape was mainly cuboid, followed by truncate-fastigate, oval, round, and kidney-shaped forms.

Overall, the high level of qualitative trait diversity observed in this collection highlights its value as a genetic resource for the conservation, utilization, and improvement of common bean adapted to Amazonian environments.

Figure 4. Histogram of 15 qualitative descriptors on 476 common bean accessions.

Relationships and variability

Cluster analysis revealed the presence of four well-defined groups among common bean (*Phaseolus vulgaris* L.) accessions from the Peruvian Amazon (Fig. 5). This clear partitioning indicates a structured pattern of morphological diversity within these landraces. Cluster formation was driven by 18 discriminant descriptors—three quantitative and 15 qualitative—that captured substantial variation in plant architecture, phenology, and seed-related traits. The observed diversity reflects not only underlying genetic differentiation but also the influence of farmer-mediated selection and adaptation to contrasting agroecological conditions. The main characteristics defining each group are summarized below.

Group 1: Short vegetative cycle and small Seed

Accessions in this group are characterized by early flowering and rapid physiological maturity, coupled with the lowest 100-seed weight among all clusters. Plants predominantly exhibit a determinate growth habit, reduced vegetative development, and relatively small leaflets, traits typically associated with early-cycle and low-input production systems.

Group 2: Large seed and climbing habit

This group comprises accessions with the largest seed size and highest seed weight. An indeterminate climbing growth habit (Type IV) predominates, characterized by long internodes and pronounced vining capacity. These accessions tend to exhibit later maturity, extended vegetative growth, and a higher number of pods per plant, reflecting their suitability for longer growing cycles and supported cropping systems.

Group 3: High yield potential and vegetative vigor

Accessions are distinguished by superior performance in key yield-related traits, including seeds per pod and total grain weight per plant. Plants display vigorous vegetative growth, dense canopy development, broad leaflets, and stems with high mechanical strength. Growth habits are mainly Type II or III, suggesting a favorable balance between vegetative biomass and reproductive allocation.

Group 4: Intermediate Morphological Types

This group includes accessions with intermediate values for quantitative descriptors. Differentiation within this cluster is primarily driven by qualitative traits, such as flower color and seed-coat patterning. These accessions appear to represent transitional or recombinant morphotypes, potentially acting as a genetic bridge between more extreme phenotypic classes.

The identified clusters were not strictly associated with geographic origin; however, certain accessions, such as Awisho and Ashpa, consistently grouped together and were predominantly cultivated in lowland environments. This pattern suggests incipient morphological differentiation linked to ecological factors, particularly altitude-related environmental gradients. Overall, the structured diversity observed among these landraces highlights their considerable potential for use in breeding programs aimed at improving yield and adaptation, while simultaneously conserving the rich agrobiodiversity of the Peruvian Amazon.

Figure 5. Tree of 476 common bean accessions according to 18 morphological descriptors

The color indicates the groups: group 1 = green, group 2 = orange, group 3 = grey, and group 4 = pink.

The first two principal components (PC1 and PC2) explained 16.9% and 11.2% of the total phenotypic variation, respectively, accounting for a cumulative 28.1% (Fig. 6). The relatively low proportion of variance captured by the first two components indicates a high level of morphological complexity among the evaluated accessions, with the remaining 71.9% of variation distributed across subsequent components. This pattern suggests that multiple traits with moderate contributions collectively shape phenotypic differentiation, rather than a few dominant variables.

The PCA biplot revealed the presence of four groups, broadly consistent with the clusters identified through hierarchical cluster analysis. However, clear separation based on morphological traits was evident only for groups 1 and 4. Group 1 was characterized by late flowering (approximately 50 days after sowing), excessive shattering of pod wall fibers, kidney-shaped and truncate–fastigiate seed forms, and a relatively high number of locules per pod (approximately 8). In contrast, group 4 comprised early-flowering accessions (approximately 38 days), strongly contracting pod wall fibers, predominantly round to oval seed shapes, and a lower number of locules per pod (approximately 5.6).

In contrast, groups 2 and 3 showed substantial overlap in the multivariate space, with no clear morphological boundaries between them. This lack of separation suggests phenotypic admixture or shared trait combinations among these groups, potentially reflecting gene flow, parallel selection

by farmers, or convergent adaptation to similar agroecological conditions. Overall, the PCA results support the cluster analysis while highlighting that only a subset of morphological traits contributes to clear phenotypic differentiation among the identified groups.

Figure 6. The two first PC biplot of 476 common bean accessions according to 18 morphological descriptors.

The color indicates the groups: group 1 = green, group 2 = orange, group 3 = grey, and group 4 = pink.

Multiple Correspondence Analysis (MCA), performed using the SNP-defined subgroups, showed that, in general, the subgroups were not distinguished by exclusive qualitative descriptors (Fig. 7). This result indicates a high degree of overlap in qualitative morphological traits among genetically defined groups. An exception was observed for subgroup 1.2, which was associated with lilac flower coloration (standard and wings), light red pod color at physiological maturity, and a carmine seed coat color. This subgroup corresponds to the Andean gene pool, suggesting that, in this case, certain qualitative descriptors are partially congruent with the genetic structure revealed by SNP data.

Figure 7. Multiple Correspondence Analysis (MCA) of 476 accessions with 15 qualitative descriptors.

The color indicates the groups and sub-groups according to the SNP-defined subgroups.

SNP marker variation and distribution

From the initial set of 665 common bean accessions, 647 presented less than 30% missing data and were retained for downstream analyses. SNP markers were filtered to retain only those with a call rate of at least 50% and a minor allele frequency (MAF) of 0.5% or higher, yielding a filtered dataset of 24,283 high-quality SNPs (Fig. 8, Table 3). Alignment to the common bean YP4 reference genome revealed that 23,050 SNPs (94.9%) were successfully mapped to chromosome positions.

For SilicoDArT markers, the applied filter of Call rate ≥ 90% and MAF of ≥ 0.5% produced a set of 29,865 markers. Additionally, alignment to YP4 genome reference revealed that 22,594 SilicoDArT markers (75.7%) were successfully mapped to chromosome positions. Results of statistical analysis were very similar than obtained with SNP markers, which is shown as supplementary material (S1-S4 Figs).

Figure 8. Histogram distribution of single nucleotide polymorphism (SNP) marker parameters

A) Callrate, B) Frequency of heterozygous genotypes, C) Read depth, D) Polymorphic Index Content (PIC), and E) Minimum allele frequency (MAF) on the filtered set of 23050 SNP markers.

Table 3. Single nucleotide polymorphism (SNP) call rate report pre and post filtering

Genetic relationships and Population Structure

Clustering analysis based on the neighbor-joining algorithm resolved the evaluated accessions into two well-defined major groups, reflecting their underlying genetic relationships (Fig. 9A). The first group comprised primarily accessions collected from the Peruvian Amazon, together with reference accessions included as an outgroup of Andean origin from the Peruvian highlands, the coastal region, and CIAT. The second group consisted mainly of Peruvian Amazon accessions, along with outgroup materials of Mesoamerican origin, including CIAT accessions and a reference sample from Mexico. This clear bipartite structure indicates that the first group corresponds to the Andean gene pool, whereas the second group represents the Mesoamerican gene pool, consistent with the recognized primary domestication centers of common bean.

Principal Coordinate Analysis (PCoA) further supported this genetic differentiation, with the first three axes explaining 62.62%, 8.97%, and 2.77% of the total molecular variation, respectively (Fig. 9B). The first axis clearly separated the Andean and Mesoamerican gene pools, while the second and third axes captured additional within-group variation. These results confirm the strong genetic structure within the collection and highlight the coexistence of both major gene pools among common bean landraces cultivated in the Peruvian Amazon.

Population structure analysis provided additional evidence for this pattern. The Evanno method identified the strongest signal of genetic structure at K = 2 (Fig. 9D), in agreement with the cluster analysis and PCoA results. These two genetic clusters correspond to the Andean and Mesoamerican gene pools and likely reflect contrasting domestication histories, evolutionary trajectories, and adaptation processes associated with their geographic origins. At a higher level of resolution (K = 4), two subgroups were detected within each major group (Fig. 9B), revealing finer-scale genetic differentiation. Notably, these subgroups showed a strong association with farmers' common names for the cultivars, providing valuable insight into the historical diffusion, local adaptation, and cropping practices of common bean in the Amazonian region (Fig. 10).

Within the Andean gene pool (Group 1), two subgroups were identified and designated as subgroup 1.1 and subgroup 1.2. Subgroup 1.1 included recently collected accessions together with reference materials from the Andean highlands (including popping beans or nuñas), and coastal regions, which were incorporated to elucidate genetic relationships among Peruvian bean germplasm. This subgroup predominantly comprised accessions known locally as Canario, Norteño, Manteca, Pinto, Chuncho, 60 Días, and Caballero. In contrast, subgroup 1.2 consisted exclusively of recently collected accessions from the Peruvian Amazon and was mainly represented by landraces referred to as Ashpa, Panamito, Ashpilla, and Vaca Paleta (Fig. 10). The genetic distinctiveness of this subgroup suggests local differentiation and adaptation under Amazonian agroecological conditions.

Similarly, the Mesoamerican gene pool (Group 2) was subdivided into subgroup 2.1 and subgroup 2.2. Subgroup 2.1 comprised exclusively recently collected Amazonian accessions, predominantly cultivated under the common names Waska, Awisho, and Poroto. Subgroup 2.2 included both Amazonian accessions and outgroup materials of Mesoamerican origin and was mainly composed of landraces known as Panamito, Shingo, Cambio 90, Pozuzo, Pinto, and Garrapata (Fig. 10). The presence of both local and reference materials within this subgroup indicates historical introductions and subsequent integration of Mesoamerican germplasm into Amazonian farming systems.

Overall, the combined clustering, PCoA, and population structure analyses reveal a complex genetic architecture of common bean landraces in the Peruvian Amazon, shaped by the coexistence of the Andean and Mesoamerican gene pools, localized diversification, and farmer-mediated selection. These findings underscore the importance of Amazonian landraces as reservoirs of genetic diversity and highlight their potential value for conservation and breeding programs targeting adaptation to diverse and changing environments.

Figure 9. Genetic structure of 647 common bean accessions with SNPs 23,050 markers.

A) Neighbor joining tree showing two main clusters, ancestry proportions from STRUCTURE analysis from K2 to K5 (left to right). B) Principal Coordinate Analysis colored according to the two main clusters of the NJ tree. C) Pie chart of the ancestry proportions of K=4 for each accession with geographic coordinates. D) Evanno plot showing the strongest signal of population structure at K=2.

Figure 10. Number of common names distribution in each subgroup of common bean from Amazonia, Peru.

Genetic Diversity (α and β) using "q" profiles

Using the two main groups identified in the Peruvian Amazon common bean panel, we quantified within-group (alpha, α) and between-group (beta, β) diversity across Hill numbers for q = 0, 1, and 2 based on the 23,050 SNP markers. The results are shown in the Table 4.

For q = 0 (allelic richness), α was 1.97 in Group1 and 1.95 in Group2, which is expected in biallelic SNPs, while β was 1.04, indicating that both groups harbor essentially the same set of allelic states with virtually no private alleles unique to either group. At q = 1 (Shannon diversity), α was identical in both groups (1.31) and β increased to 1.20, which indicates that although within-group allele-frequency balance is similar, the groups begin to differ in how common those alleles are between populations. At q = 2 (Gini–Simpson/expected heterozygosity), α was 1.22 in Group1 and 1.23 in Group2, whereas β rose to 1.4–1.5, indicating a clearer differentiation driven primarily by shifts in the frequencies of common alleles.

Taken together, these q-profiles show minimal presence/absence differentiation but progressively stronger frequency-based divergence from q = 0 to q = 2, indicating that the genetic structuring between groups is explained more by differences in the prevalence of shared alleles than by unique allelic repertoires.

Table 4. Hill number of α (intrapopulation) and β (interpopulation) diversity indices according to the 23,050 loci SNP and two groups of common beans from Amazonia, Peru.

Using the same 23,050 SNPs, we extended the q-profile analysis to four populations defined by previous cluster and STRUCTURE analysis. The results of the analysis in the four subgroups are shown in Table 5.

For q = 0, α ranged narrowly from 1.82 to 1.94 (see Table 5), indicating that all populations harbor nearly the full set of biallelic states with only minor differences in allelic richness. At q = 1 , α values differentiated the populations more clearly (Subgroup1_1 = 1.31; Subgroup1_2 = 1.25; Subgroup2_1 = 1.16; Subgroup2_2 = 1.30), which indicates varying degrees of allele frequency balance: Subgroup1_1 and Subgroup2_2 exhibit more even allele frequencies, whereas Subgroup2_1 shows greater skew toward common alleles. At q = 2, the ranking persisted (Subgroup2_1 = 1.10; Subgroup1_2 = 1.16; Subgroup2_2 = 1.21; Subgroup1_1 = 1.22), indicating that differences among populations are driven primarily by the frequencies of common alleles.

Taken together, the four-way comparison shows minimal differences in allelic repertoires (q = 0) but increasing divergence at q = 1 and q = 2, which indicates that genetic differentiation among these subpopulations is explained less by presence/absence of allelic states and more by shifts in the prevalence and balance of shared alleles.

Comparing the two subgroups within each main group we observe little presence/absence differentiation but a clear frequency-based divergence. For subgroup1_1 versus subgroup1_2, β increased from 1.06 at q = 0 to 1.24 at q = 1 and ~1.29 at q = 2, which indicates that while both subgroups share essentially the same allelic states, they differ modestly to moderately in allele-frequency balance, with the strongest contrast driven by common alleles. A similar behavior is observed for subgroup2_1 versus subgroup2_2 (Table 5).

Table 5. This is the Table 5 Title. Hill number of α (intrapopulation) and β (interpopulation) diversity indices according to the 23,050 loci SNP and four subgroups of common beans from Amazonia, Peru.

Population Heterozygosity

The parameters of diversity using heterozygosity, according to the 23,050 loci SNP, two-groups and four subgroups of common beans are shown in the Table 6.

Two-groups analysis

Group 1: where the effective sample size was ~240 individuals, showing 22,281 polymorphic loci (almost all, 97% of the total). The Ho = 0.041, meaning that on average, ~4.1% of the loci per individual are heterozygous. Then, the He/uHe = ~0.148, under Hardy-Weinberg Equilibrium (HWE) shows around ~14.8% of heterozygous loci would be expected. The FIS = 0.52 (inbreeding coefficient), indicated a severe heterozygote deficit. The population is less heterozygous than expected, which reflect inbreeding, that is a expected result in a self-pollinating populations as a common bean.

Group 2: The effective sample size was ~318 individuals, showing 21,846 polymorphic loci (94.8% of the total). The Ho = 0.035, around ~3.5% of loci are heterozygous on average, even lower than group 1. The proportion He/uHe= ~0.147 is similar to group 1 in expected heterozygosity; and FIS= 0.54, also shows strong heterozygote deficit, very comparable to group 1. Comparing both Group 1 vs. Group 2, they show very similar He (~0.15), indicating that the allele pool and frequencies are similar. Ho is lower in group 2 than group 1(0.035 vs. 0.041), suggesting that individuals in group 2 express less heterozygosity. The FIS is elevated in both populations (>0.5), reinforcing the idea of a generalized heterozygote deficit, which is expected for common bean populations. Finally, group2 has more monomorphic loci than group 1 (1,204 vs 769), which could reflect loss of variation or fixation at certain sites.

Four subgroups analysis

The subgroup1_1 has effective size ~136 individuals and 21,511 polymorphic loci (93% of the total). The Ho = 0.032, quite low compared to He = 0.145; and the FIS = 0.58, reflecting a strong heterozygote deficit.

The subgroup 1_2 has effective size ~104 individuals and 20,444 polymorphic loci (88.7%), more monomorphic loci than subgroup1_1. The Ho = 0.053, higher than subgroup1_1, individuals show slightly more heterozygosity. However, the He = 0.115 was lower than subgroup1_1; and the FIS = 0.35, still positive but lower heterozygote deficit than in subgroup1_1.

Subgroup2_1 has effective size ~131 individuals and 18,877 polymorphic loci (82%), the lowest of the four subgroups. Also, the Ho = 0.029 and He = 0.071 were the lowest of all subgroups, indicating a loss of diversity; and the FIS = 0.30 showed a moderate heterozygote deficiency.

Subgroup2_2 has effective size ~187 individuals and 21,563 polymorphic loci (93.5%), comparable to subgroup1_1. The Ho = 0.040 showed an intermediate value between subgroup1_1 and subgroup1_2; and the He = 0.139 was intermediate too; and FIS = 0.53, indicating a severe heterozygote deficiency.

Comparisons between subgroups within the group.

Group_1: the subgroup1_1 has a higher He (0.145) but a lower Ho, resulting in a very high FIS (0.58). While, the subgroup1_2 has a lower He (0.115) but a relatively higher Ho (0.053), which reduce the deficit of heterozygosity (FIS = 0.35). The subgroup1_1 appears more structured as an self-pollinating population, while subgroup1_2 maintains more heterozygotes despite lower overall diversity.

Group 2: the subgroup2_1 has the lowest diversity among the four subgroups (Ho = 0.029; He = 0.071). However, the subgroup2_2 recovers diversity (He = 0.139), but its FIS is still high (0.53). Within Group_2, there is a clear inequality; subgroup2_1 present loss of diversity; and subgroup2_2 is more diverse but lacks heterozygotes.

In all four subgroups, there is a clear disconnection between Ho and He, all subgroups have fewer heterozygotes than expected under Hardy–Weinberg equilibrium. Also, the positive and high FIS (0.30–0.58) in all subgroups suggest inbreeding, internal structure favoring homozygotes, which is expected in common bean.

Table 6. This is the Table 6 Title. Genetic diversity parameters within groups and subgroups with the 23,050 loci SNP of common beans from Amazonia, Peru.

This is the Table 6 legend. n.Ind (ef.) = effective sample size; n.Loc = number of loci; polyLoc = number of polymorphic loci; monoLoc = monomorphic loci; Ho (Observed heterozygosity; proportion of heterozygous loci per individual, average across the population); He (Expected heterozygosity, Nei 1978; expected heterozygosity under Hardy–Weinberg equilibrium); uHe (unbiased He, corrected for sample size); FIS (Inbreeding coefficient = 1 – Ho / uHe, positive values indicate a deficit of heterozygotes (possible inbreeding or structure) and negative values indicate an excess).

Discussion

Morphologic variability

The morphologic diversity observed in term of type of plant, flowering time, color, size, form of pods and seeds confirms that Amazonian *P. vulgaris* represents an important genetic reservoir. The presence of distinct genetic clusters suggests that both environmental heterogeneity and farmer practices shape phenotypic variability. Similar levels of morphological diversity have been reported in Brazil [42], Mexico [43], Spain [44], Nigeria [45], Pakistan [46], Ethiopia [47]. In Peru, however, most previous studies have focused on highland germplasm, using small sample size [23] [22] [21] [48] and lately sample from Amazonia [25]; which are not representative to allow a comprehensive estimation of common bean variability. Our results, show that much of the morphological diversity is conserved on farms, where farmers save, select, and replant seeds year after year, ensuring both autonomy and household income [49] [50]. In addition, frequent seed acquisition by farmers from traditional markets further promotes gene flow and admixture, particularly relevant under tropical conditions where high temperature and humidity shorten seed longevity, and its storage challenges; causes rapid loss of viability. Together, these practices contribute to the maintenance and renewal of agrobiodiversity in the Peruvian Amazon. It is relevant to highlight the farmer behaviors, who collet new material and sow all together with own local landraces. Then, the agroecological mosaic, coupled with traditional knowledge, has led to the maintenance of a broad range of landraces with varying morphological traits. A large portion of this agrobiodiversity is still conserved *in situ*, within Indigenous and local farming systems. Farmers contribute to the conservation and evolution of these cultivars by using and exchanging seeds year by year [51] [52]. Additionally, field observations and interviews revealed that many farmers regularly acquire seeds from traditional markets to replace planting stock each season. This behavior is likely influenced by tropical environmental constraints—such as high temperatures and humidity—which accelerate seed aging and reduce viability during storage. Thus, constant renewal of planting material becomes a necessary strategy. The morphological diversity recorded—particularly in traits like growth habit, seed color, and pod characteristics—supports the idea of local adaptation and cultural preferences influencing varietal selection. Morphological characterization, though often considered basic, remains a valuable and accessible tool for identifying distinct genotypes and understanding their agronomic potential and farmer preferences.

Molecular diversity

SNP-based analyses, cluster and Principal Coordinate analysis (PcoA) revealed that the accessions were assigned into two distinct groups, corresponding to the Andean or Mesoamerican gene pools,

284 and 363 accessions, respectively (Fig. 9). Of the Amazonia accessions, 56.1 % clustered with Mesoamerican pool and 43.9 % with the Andean gene pools. However, this tendency was largest in others common bean germplasm genetic diversity studies, e.g. in Brazil was reported, more than 70 % of Mesoamerican gene pool, [4]; [53]; and in Ethiopian germplasm, the 284 common bean accessions were roughly classified into Andean and Mesoamerican gene pools, assigning 188 (65.05%) accessions in the Mesoamerican group, while the remaining 96 (33.22%) accessions were classified in the Andean gene pool [28]. The population structure analysis (based on K = 2 groups) showed that the common bean accessions from Peruvian amazon were distributed between two gene pools, Andean and Mesoamerican with a low degree of admixture. By comparison, when considering K = 4, both the Andean and Mesoamerican gene pool was clustered in two subgroups, with low admixture.

This structure is consistent with findings from USDA core collections [54], where PCoA and NJ trees confirmed the Andean–Mesoamerican split and further subdivisions into ecogeographic races. These results indicate that Amazonian germplasm is integrated into the global diversity framework of *P. vulgaris* while retaining unique local substructure. However, in Brazilian germplasm genetic diversity studies in common bean were obtained more diversity in Mesoamerican gene pool using GBS analysis [53]. In overall, the genetic variation detected through DarTseq SNP analysis showed that, the clustering agrees with that obtained by the principal coordinate analysis and population structure analyses.

The high genetic variation detected through DArTseq SNP analysis complements the phenotypic findings and highlights the importance of both *in situ* and *ex situ* conservation. Many of these accessions, particularly those maintained only on-farm, are at risk of being lost due to changes in land use, market pressures, climate change, or generational shifts in farming practices. Comprehensive genetic characterization enables the identification of unique and underrepresented alleles, which can be prioritized for conservation and potentially incorporated into breeding programs aimed at improving resilience to biotic and abiotic stresses.

Alpha (α) and Beta (β) Genetic Diversity

The Peruvian Amazonia common beans, according to the alpha-diversity estimates showed similar internal variability in both gene pools, with most loci being biallelic and rare alleles contributing little or marginal to overall diversity. Beta-diversity revealed that Andean and Mesoamerican groups largely share the same alleles but differ in allele frequencies, reflecting the combined effects of local selection, drift, and demographic histories.

The four subgroups identified at K=4 maintained nearly complete allelic richness, with differentiation arising mainly from differences in allele frequencies rather than unique variants. This pattern is consistent with historical farmer migration from the Andes into Amazonian areas, where likely Andean beans were introduced and later mixed with locally adapted materials, followed by continuous selection and cultivation under tropical conditions. This result is similar what was found by [55]on diversification and population structure in common beans, suggesting geographic isolation, founder effects or natural selection and introgression were involved in creating the diversity of cultivated beans.

Population Heterozygosity

Despite the high proportion of polymorphic *loci*, observed heterozygosity (Ho ≈ 0.035–0.041) was markedly lower than expected (He ≈ 0.147–0.148). Positive and high inbreeding coefficients (FIS = 0.30–0.58) across all groups and subgroups indicate strong homozygosity, which is expected in a predominantly self-pollinating crop such as common bean. Similar patterns of heterozygote deficit have been reported in Brazil and Ethiopia [42] [28].

In both morphological and molecular characterization, no duplicates were found in the material under study, this result is similar that was found evaluating 67 accessions based on morphological traits and molecular markers from Brazil [42]; which suggests the importance of keeping all accessions in the germplasm bank since they represent a valuable source of genetic diversity. Importantly, this variability has remained largely unexploited in breeding programs, which have focused on disease resistance; lagging to obtain high yielding and high-quality cultivars in major production regions [56] [57] Incorporating Amazonian diversity into breeding pipelines could broaden the adaptive base of common bean. Hence, the conservation and systematic use of this germplasm are of strategic importance for Peru national genetic resources and future crop improvement.

Conclusion

This article addresses the under characterization of genetic diversity in common bean (landraces from the Peruvian Amazon, which limits their use in breeding programs. Prior to this study, most research focused on commercial cultivars from highland and coastal regions, neglecting the unique genetic resources found in the Amazon. This study demonstrates that common bean landraces from the Peruvian Amazon harbor remarkable morphological and genetic diversity, confirming the region as a key reservoir of agrobiodiversity. Both morphological and SNP-based analyses consistently revealed two major groups corresponding to the Andean and Mesoamerican gene pools, each subdivided into local subgroups that include unique Amazonian accessions.

There was high allelic richness but low observed heterozygosity, consistent with the self-pollinating nature of the species. Furthermore, genetic diversity analyses showed similar internal variability between groups, with differences arising mainly from allele frequency rather than different allele presence, and confirmed the expected high levels of inbreeding in this predominantly self-pollinated crop. Importantly, no redundant accessions were detected, highlighting the unique value of each collected sample.

The integration of morphological descriptors with DArTseq SNP markers proved effective in identifying structure and patterns of variation, demonstrating the complementarity of these approaches for germplasm management. Because much of this diversity is maintained only *in situ* by local farmers, there is an urgent need to reinforce both *in situ* and *ex situ* conservation strategies.

This study enhances our understanding of the genetic diversity present in Amazonian common beans, highlighting the need for conservation strategies to preserve these valuable resources for future breeding efforts. They provide novel opportunities for breeding programs seeking traits related to tropical adaptation, resilience to climatic stresses, and farmer- and market-preferred characteristics. Harnessing this diversity is essential to ensure food security and sustainable agriculture development in the face of future challenges.

Acknowledgements

The authors are thankful to SAGA- Genetic Analysis Service for Agriculture, CIMMYT-Mexico; for their support for genotyping. Also, we thank local farmers for their cooperation and the research institutions (UNALM, UNSM, UNU) that supported this study.

Author contributions

JT, MB, DV and MP contributed to conceptualization, to methodology and to writing; JP, JC and YV contributed to investigation; DV, RR and CP to data curation, writing—original draft preparation, visualization, and formal analysis; JS contributed to writing—review; JE contributed to review; RO contributed to writing—review and editing; RB contributed to conceptualization, to methodology to supervision and to funding acquisition. All authors have read and agreed to the published version of the manuscript.

Conflicts of interest

The authors declare no conflict of interest.

Ethical approval

This article does not contain any studies with human participants and animals and ethical approval is not applicable.

Data availability

Supplementary Tables 1 and 2 provide the data on morphological characterization and DArTseq single nucleotide polymorphisms. They are available at

Blas, Raul, 2026, "Morphological characterization data of common bean_Amazonia_Peru", https://doi.org/10.17026/LS/6IRCBV, DANS Data Station Life Sciences, DRAFT VERSION

Blas, Raul, 2026, "DArTseq_SNP_647x23050 markers_common_bean_Peru", https://doi.org/10.17026/LS/SWIWOJ, DANS Data Station Life Sciences, DRAFT VERSION

References

1. Wang Y, Xiaopeng H, Chunhai C, Haigang W, Peng G, Xukui Y, et al. Telomere-to-telomere genome of common bean (*Phaseolus vulgaris* L.) YP4. 2025; GigaScience 14: giaf001. Doi: 10.1093/gigascience/giaf001

2. Bitocchi E, Nanni L, Bellucci E, Rossi M, Giardini A, Zeuli PS, et al. Mesoamerican origin of the common bean (*Phaseolus vulgaris* L.) is revealed by sequence data. Agricultural Sciences. 2012; 109: 788–796. Doi: 10.1073/pnas.1108973109

3. Kwak MKJAGP. The putative Mesoamerican domestication center of *Phaseolus vulgaris* is located in the Lerma-Santiago Basin of Mexico. Crop Science. 2009; 49: 554–563.

4. Bellucci E, Bitocchi E, Rau, D, Rodriguez M, et al. Genomics of origin, domestication and evolution of *Phaseolus vulgaris*. In: Tuberosa, R, de, Graner A, Frison E (eds). Springer, Dordrecht, The Netherlands. 2014. Pp. 483–507. Doi: 10.1007/978-94-007-7572-5_20

5. Frascarelli G, Galise T, D'Agostino N, Cafasso D, Cozzolino S, Cortinovis G, et al. The evolutionary history of the common bean (*Phaseolus vulgaris*) revealed by chloroplast and nuclear genomes analysis. Theoretical and Applied Genetics. 2025; 138: 47. Doi: 10.1007/s00122-025-04832-z

6. Singh SP. Broadening the genetic base of common bean cultivars. Crop Science. 2001; 41: 1659–1675. Doi: 10.2135/cropsci2001.1659

7. Gepts P. Origin and evolution of common bean: past events and recent trends. HortScience. 1998; 33: 1124–1130.

8. Schmutz JMP, Wu, GA, Cannon SB, Grimwood J, Jenkins J, Shu S, et al. A reference genome for common bean and genome-wide analysis of dual domestications. Nature Genetics. 2014 ;46: 707–713, 7. Doi: 10.1038/ng.3008

9. Angioi SA, Rau D, Attene G, Nanni L, Bellucci E, Logozzo G, et al. Beans in Europe: origin and structure of the European landraces. Theoretical and Applied Genetics. 2010; 121: 829–843.

10. Paredes M, Becerra V, Tay J. Inorganic nutritional composition of common bean (*Phaseolus vulgaris* L.) genotypes race Chile. Chilean Journal of Agricultural Research. 2009; 69: 486–495.

11. Suárez-Martinez SE, Ferriz-Martínez RA, Campos-Vega R, Elton-Puente JE. Bean seeds: leading nutraceutical source for human health. Journal of Food. 2016; 14: 131–137.

12. Jensen ES, Peoples MB, Boddey RM, Gresshoff PM, Hauggaard-Nielsen H, Alves BJR, et al. Legumes for mitigation of climate change and the provision of feedstock for biofuels and biorefineries. A review. Agronomy for Sustainable Development. 2012; 32: 329–364. Doi: 10.1007/s13593-011-0056-7

13. United Nations. International Year of Pulses, 2016. UN Resolution68/231. 2014. 68th Session General Assembly, New York. https://docs.un.org/en/A/RES/68/231

14. Saburido M, Hererra A. El frijol en la era genómica. Revista Digital Universitaria. 2015; 2: 16.

15. Calero-Hurtado A, Quintero-Rodríguez E, Olivera-Viciedo D, Pérez-Díaz Y, Castro-Lizazo I, Jiménez J, et al. Respuesta de dos cultivares de frijol común a la aplicación foliar de microorganismos eficientes. Cultivos Tropicales. 2018;39: 5-10. Available from: http://scielo.sld.cu/scielo.php?script=sci_arttext&pid=S0258-59362018000300001&lng=es&tlng=pt

16. FAO Legumbres. Pequeñas semillas, grandes soluciones. Food and Agriculture Oganization of the United Nations, Rome, Italy. 2018; 292.

17. Ministerio de Agricultura y Desarrollo Rural, «El agro en cifras. MIDAGRI. 2024. Available: https://siea.midagri.gob.pe/portal/phocadownload/datos_estadisticas/mensual/Agro/2024/Agro_en_cifras%2006%202024.pdf

18. Genesys. Genesys Global Portal on Plant Genetic Resources. 2021. Available: www.genesys-prg.org.

19. I.N.I.A. Catálogo de ñuña en el banco de germoplasma del INIA. 2021. Available: https://hdl.handle.net/20.500.12955/2112

20. I.N.I.A. Catálogo de frijol en regiones Andinas del banco de germoplasma del INIA. 2023. Available: https://hdl.handle.net/20.500.12955/1404

21. Balarezo JC. Evaluación Agromorfológica Y Caracterización Molecular de la ñuña (*Phaseolus vulgaris* L. Idesia (Arica). 2009;27: 29-40. doi: 10.4067/S0718-34292009000100005

22. Marmolejo K. Variabilidad genética del frijol común tipo ñuña (*Phaseolus vulgaris* L.) en las localidades de Carhuaz y Chiquián, Ancash. M.Sc. Thesis. Universidad Nacional Agraria La Molina. 2018. Available: http://repositorio.lamolina.edu.pe/handle/UNALM/3398

23. Amaringo S. Caracterización fenotípica de 5 ecotipos de frijol común (*Phaseolus vulgaris* L.) en el fundo Aucaloma – Lamas. BSc. Thesis. Universidad Nacional de San Martín. Tarapoto; 2015. Available: http://hdl.handle.net/11458/635

24. Collado LA. Diversidad cultivada y socio-cultural en la Amazonia central del Perú. M.Sc. Thesis. Universidad Nacional Agraria de la Selva. 2002. Available: http://repositorio.unas.edu.pe/handle/UNAS/900

25. Vásquez J, Vilca-Valqui NC, Malqui R, Fernández E, Duarez E, Ayala R. Agromorphological characterization of *Phaseolus* spp., accessions in Amazonas region, Peru. Bioagro. 2024;36: 129–142. Doi: 10.51372/bioagro362.1

26. Seo E, Kim K, Jun T, Choi J, Kim S, Sun H. Population structure and genetic diversity in Korean cowpea germplasm based on SNP markers. Plants. 2020; 9: 1190. Doi: 10.3390/plants9091190

27. Verma S, Gupta S, Bandhiwal N, Kumar T, Bharadwaj C, Bhatia S. High-density linkage map construction and mapping of seed trait QTLs in chickpea (*Cicer arietinum* L.) using genotyping-by- sequencing (GBS). Scientific Reports. 2015 ;5: 17512. Doi: 10.1038/srep17512

28. Gelaw YM, Eleblu JSY, Ofori K, Fenta BA, Mukankusi C, Emam EA, et al. High-density DArTSeq

SNP markers revealed wide genetic diversity and structured population in common bean (*Phaseolus vulgaris* L.) germplasm in Ethiopia. Molecular Biology Reporter. 2023;50: 6739-6751. doi: 10.1007/s11033-023-08498-y

29. International Board for Plant Genetic Resources (IBPGR), *Phaseolus vulgaris* descriptors. International Board for Plant Genetic Resources, Rome, Italy. 1982:32.

30. Team RC. R: a language and environment for statistical computing. Vienna, Austria: R Foundation for Statistical Computing; 2025. Available: https://www.R-project.org/

31. Posit team, RStudio: Integrated Development Environment for R. PBC, Boston, MA: Posit Software; 2025. Available: http://www.posit.co/

32. Le S, Josse J, Husson F. FactoMineR: An R package for multivariate analysis. Journal of Statistical Software. 2008; 25: 1-18. Doi: 10.18637/jss.v025.i01

33. Kassambara A, Mundt F. Factoextra: extract and visualize the results of multivariate data analyses. 2020. Doi: 10.32614/CRAN.package.factoextra

34. Wickham H. ggplot2: Elegant graphics for data analysis. Springer-Verlag New York; 2016.

35. Liaw A, Wiener M. Classification and Regression by randomForest. R News. 2002;2: 18–22. Available: https://CRAN.R-project.org/doc/Rnews/

36. Sansaloni C, Petroli C, Jaccoud D, Carling J, Detering F, Grattapaglia D, et al. Diversity Arrays Technology (DArT) and next-generation sequencing combined: genome-wide, high throughput, highly informative genotyping for molecular breeding of Eucalyptus. BMC Proceedings. 2011;5(P54). doi: 10.1186/1753-6561-5-S7-P54

37. Kilian A, Wenzl P, Huttner E, Carling J, Xia L, Blois H., et al. Diversity Arrays Technology: A generic genome profiling technology on open platforms. In: Pompanon F, Bonin A, editors. Data Production and Analysis in Population Genomics. Doi: 10.1007/978-1-61779-870-2_5

38. Petroli C, Kilian A. Introduction to the DArTseq genotyping method and its data outputs. CIMMYT Research Data & Software Repository Network. 2019; v1. Available: https://hdl.handle.net/11529/10548358/

39. Gruber B, Unmack PJ, Berry OF, Georges A. dartr: An Rpackage to facilitate analysis of SNP data generated from reduced representation genome sequencing. Molecular ecology resources. 2018; 18: 691–699.

40. Perrier X, Jacquemoud-Collet JP. DARwin software: Dissimilarity analysis and representation for Windows. 2006. Available: https://darwin.cirad.fr/

41. Raj A, Stephens M, Pritchard JK. fastSTRUCTURE: variational inference of population structure in large SNP data sets. Genetics. 2014;197: 573–589.

42. Paula E, Almeida RN, O. Santos T, Souza Neto JD, Riva-Souza EM, Posse SCP, et al. Genetic Diversity of common bean (*Phaseolus vulgaris* L.) landraces based on morphological traits and

molecular markers, Plants. 2024;13: 2584. Doi: 10.3390/plants13182584

43. García-Narváez AL, Hernández-Delgado S, Hernández-Delgado JL, Mayek-Pérez N, Morphological and agronomic variability of bean germplasm cultivated in Oaxaca, México. Revista bio ciencias. 2020; 7.

44. Arteaga S, Yabor L, Torres J, Solbes E, Muñoz E, Díez, MJ. Morphological and Agronomic Characterization of Spanish Landraces of *Phaseolus vulgaris* L. Agriculture. 2019; 9(7):149.

45. Kouam E, Kamga-Fotso A-A, Anoumaa M. Exploring agro-morphological profiles of *Phaseolus vulgaris* germplasm shows manifest diversity and opportunities for genetic improvement. Journal of Agriculture and Food Research. 2023;14. Doi: 10.1016/j.jafr.2023.100772

46. Nawaz I, Zeb T, Zeb SB, Sherani, J. Evaluation of common beans (*Phaseolus vulgaris* L.) land races based on qualitative traits derived from Himalayan, Andean and Mesoamerican regions. Sarhad Journal of Agriculture. 2022; 38(1):275–286.

47. Tigist SG, Sibiya J, Amelework A, Keneni G. Agromorphological and physiological performance of ethiopian common bean (*Phaseolus vulgaris* L.) genotypes under different agroecological conditions. Plants. 2023;12(12): 2342. https://doi.org/10.3390/plants12122342

48. Castro R, Quispe D. Caracterización morfológica de genotipos de frijol (*Phaseolus vulgaris* L.) en San Juan de Uchubamba-Jauja, La Merced Perú. BSc. Thesis. Universidad Nacional Daniel Alcides Carrión. 2017. Repositorio Universidad Nacional Daniel Alcides Carrión (UNDAC).

49. Figueiredo Menezes Cavalcanti, T., Pombo Sudré, C., Wesley da Silva Corrêa, J., dos Santos Bento, C., Knoblauch Viega de Andrade, E., Kazue Nakamura Fukuji, K., Rodrigues, R. Custodians of common bean diversity in Rio de Janeiro state, Brazil: revealing their socioeconomic and environmental profile. Agroecology and Sustainable Food Systems. 2021; 45: 1165–1188. Doi: 10.1080/21683565.2021.1888186

50. de Avila J, Coelho F, Sardo P, Haddade I, Moonen A. In situ conservation of agrobiodiversity carried out by traditional family farmers in a mountainous region of Brazil. Agroecology and Sustainable Food Systems. 2024; 49(6):797–820, Doi: 10.1080/21683565.2024.2445729

51. Castañeda-Álvarez NP, Haan S, Juárez H, Khoury CK, Achicanoy HA, Sosa CC, et al. Ex situ conservation priorities for the wild relatives of potato (*Solanum* L. Section Petota). PLoS ONE. 2015; 10: 0122599. Doi: 10.1371/journal.pone.0122599

52. Bellon MR Planting hybrids, keeping landraces: agricultural modernization and tradition among small-scale maize farmers in Chiapas, Mexico. World Development. 2011; 39(8):1434–1443. Doi: 10.1016/j.worlddev.2010.12.010.

53. Elias J, Gonçalves-Vidigal M, Ariani A, Valentini G, Martiniano-Souza M, Bisneta MV, et al. Genome-environment association analysis for bio-climatic variables in common bean (*Phaseolus vulgaris* L.) from Brazil. Plants. 2021; 10:1572. Doi: 10.3390/plants10081572

54. Kuzay S. Is the USDA core collection of common bean representative of genetic diversity of the species, as assessed by SNP diversity. *Crop Science*. 2020;60: 1398–1414. Doi:

10.1002/csc2.20032

55. Blair MW, Soler A, Cortés, AJ. Diversification and population structure in common beans (*Phaseolus vulgaris* L.). PLoS ONE. 2012; 7(11): e49488. doi: 10.1371/journal.pone.0049488

56. Singh SP, Schwartz HF, «Breeding common bean for resistance to diseases: a review. Crop Science. 2010; 50: 2199–2223. Doi: 10.2135/cropsci2009.03.0163.

57. Cichy KA, Porch TG, Beaver JS, Cregan P, Fourie D, Glahn, RP, et al. A Phaseolus vulgaris Diversity Panel for Andean Bean Improvement. Crop Science. 2015; 55:2149–2160. Doi: 10.2135/cropsci2014.09.0653

Supporting information

Figure S1 Histogram distribution of SilicoDArT marker parameters

A) Callrate, B) OneRatio, C) Polymorphic Index Content (PIC), and E) minimum allele frequency (MAF) on the initial filtered ser 29865 SNP markers.

Figure S2. Dendrogram of 647 accessions of common bean, according to the 29865 SilicoDArT markers.

Figure S3. Principal coordinate analysis (PCoA) of 647 accessions of common bean according to SilicoDArT markers.

Figure S4. Population structure of 647 accessions of common bean with SNP-DArTseq markers.

K2 to K5 (from left to right)

Journal Requirements:

1. Please ensure that your manuscript meets PLOS ONE's style requirements, including those for file naming. The PLOS ONE style templates can be found at…

The manuscript was adapted to the template

2. In your Methods section, please provide additional information regarding the permits you obtained for the work. Please ensure you have included the full name of the authority that approved the field site access and, if no permits were required, a brief statement explaining why.

It is included the file of permission on genetic resources accessions from Peruvian authority (INIA), which was not required to develop the research.

3. Thank you for stating the following in the Acknowledgments Section of your manuscript:

4. We note that Figures 1 and 11 in your submission contain [map/satellite] images which may be copyrighted. All PLOS content is published under the Creative Commons Attribution License (CC BY 4.0),…

The maps were elaborate using R program, according the following methodological approach for creating the map of beens accessions in Software R consisted of the following sequence: 1) obtaining elevation data (DEM) for the Peruvian territory using the get_elev_raster() function from the elevatr package; 2) loading political boundary vector layers via rnaturalearth; 3) importing and converting collected bean occurrence points—stored in clipboard data—into a spatial format (sf); and 4) integrating all layers into a two-dimensional map with ggplot2, overlaying the shaded relief (elevation raster with a customized color palette), departmental borders, collection locations and cartographic elements such as a north arrow and scale bar. Finally, the map was exported as a TIF image.

Additional Editor Comments:

Since authors have genotypic data and phenotypic data on multiple traits, I suggest to run the GWAS to identify genomic regions associated with traits.
Figure quality need to be improved. increase font size in Fig 6, 7 and 12.

This GWAS analysis will be done for another paper which should include multilocal agronomic evaluation and nutritional analysis.
Figures quality were improved according to the journal recommendations.

Reviewers' comments:

5. Review Comments to the Author

Please use the space provided to explain your answers to the questions above. You may also include additional comments for the author, including concerns about dual publication, research ethics, or publication ethics. (Please upload your review as an attachment if it exceeds 20,000 characters)

Reviewer #1: Page 9: a. Kindly include the aim of study

It was added the aim: “For this reason, the main aim of this study was to assess the genetic diversity of *P. vulgaris* populations.”

b. I believe 667 accessions were collected but only 476 were analysed. Please correct this this number in the abstract, materials and method and result section.

The reason of discordance number, it was already indicated in page 9 (Morphological variability)

We describe the characterization of 476 accessions (from 667 sowed), the rest of 191 accessions did not progress in the field trial.

c. Kindly expand the result section to strengthen the abstract. The information on population structure and clustering is not clearly highlighted, and much of the rich detail appears to be hidden.
It was expanded.

Kindly refer to the attached manuscript for my additional comments. Thank you. Excellent write up.

Reviewer #2: The study entitled “Population structure and diversity of common bean (Phaseolus vulgaris L.) landraces in the Peruvian Amazon” provides interesting advances in understanding the population structure and genetic diversity of common bean landraces collected from different region by utilizing both morphological and SNP markers information. However, the manuscript, in its current form, requires substantial revision before it can be considered for publication, most especially in mythology, results presentation, and conclusion. Below is the specific point to be addressed:
1. The Materials and Methods section lack essential details. Critical information such as descriptions of the recorded traits and the procedures used for data processing and analysis were insufficiently described. To ensure the reproducibility of this study, please provide a detailed description of each studied trait, rather than only indicating the number of colors or types.

It was improved the explanation on methodology:

Data was recorded as following for each descriptor, taken into consideration common bean descriptors chart:
Growth habit: Determined based on the description of growth habits and reference diagrams in the descriptor.
Days to flowering: Number of days elapsed from sowing until the opening of the first floral bud in at least 50% of the evaluated plants.
Color of standard: Observation on freshly opened flowers from the inflorescence at the fourth node on each evaluated plant, according to the descriptor color chart.
Wing color: Observation on freshly opened flowers from the inflorescence at the fourth node on each evaluated plant, according to the descriptor color chart.
Green pod color: Observation on 10 fully developed immature pods, according to the descriptor color chart.
Pod length: Average length (cm) of 10 pods obtained from five plants.
Pod cross-section: Transverse section of 10 fully developed immature pods at the level of the second seed from the distal end, according to the descriptor description chart.
Pod curvature: Observation of 10 fully developed immature pods, according to the descriptor description chart.
Number of locules: Average of number of locules from longest pod on10 pods obtained from five plants.
Pod wall fiber: Observation on 10 pods obtained from five plants, according to the descriptor model.
Pod apex position: Observation of 10 pods obtained from five plants, according to the descriptor model.
Pod apex orientation: Observation of 10 pods obtained from five plants, according to the descriptor model.
Pod color at the physiological maturity: Observation of 10 dry pods at the harvesting time, according to the descriptor color chart.
Seed coat pattern: Observation of 10 seeds, taken from the seed closest to the apex of each pod, according to the descriptor pattern chart.
Seed coat lighter color: Observation of primary (light) seed coat color on 10 seeds, taken from the seed closest to the apex of each pod, according to the descriptor color chart.
Seed coat darker color: Observation of secondary (background) seed coat color on 10 seeds, taken from the seed closest to the apex of each pod, according to the descriptor color chart.
Seed brightness: Observation of seed coat luster on10 seeds, taken from the seed closest to the apex of each pod.
Seed shape: Observation of 10 seeds, taken from the middle of each pod.

Prior to data analysis, it was estimated the average for the quantitative data and recorded the mode for the qualitative data (the value that appears most frequently in the dataset, representing the most common category of descriptors). Then, the matrix was used in the univariate and multivariate analysis.

…..
2. The Results section primarily restates elements of the methodology and discussion rather than presenting the main findings in detail, and the figure quality could be improved to allow proper evaluation

Figures' quality was improved.

3. "Despite its importance, most studies on common bean in Peru have focused on commercial varieties grown in the highlands and coastal regions, emphasizing agronomic practices and genetic improvement using both local and introduced varieties". Please provide some references that have investigated these areas.

There is no specific reference about the following paragraph, it was written taking into consideration our comprehensive bibliographic revision on common beans in Peru and our knowledge on research activities at the UNALM Legume Program, … "in Peru have focused on commercial varieties grown in the highlands and coastal regions, emphasizing agronomic practices and genetic improvement using both local and introduced varieties"

4. The conclusion provides only a summary of the results and does not effectively address the research objective

This article addresses the under characterization of genetic diversity in common bean (landraces from the Peruvian Amazon, which limits their use in breeding programs. Prior to this study, most research focused on commercial cultivars from highland and coastal regions, neglecting the unique genetic resources found in the Amazon. The comprehensive evaluation of 476 accessions using both phenotypic and genomic approaches found two major genetic groups corresponding to Andean and Mesoamerican gene pools, each with distinct subgroups. There was high allelic richness but low observed heterozygosity, consistent with the self-pollinating nature of the species. This study enhances our understanding of the genetic diversity present in Amazonian common beans, highlighting the need for conservation strategies to preserve these valuable resources for future breeding efforts.

5. "Cluster analysis based on morphological traits grouped the accessions into four major clusters

In was corrected.

6. (Figure 3)." But Figure 3 showed Boxplot of quantitative descriptors, please verify and indicate the correct figure

It was corrected; it is figure 5.

7. Although the data were said to be filtered at MAF of ≥ 1%, the MAF distribution extended to 0.5 (Figure 8E). Please clarify this discrepancy

It is 0.5%, already corrected.

In addition to the comment above, the following suggestion would enrich the manuscript

1. Since the study was conducted at a single location, in a non-replicated trial with no appropriate experimental design, the quantitative traits may be misleading (i.e., the observed differences might not reflect the true genetic merit of the landraces under investigation). Therefore, it is recommended to include qualitative descriptors in the clustering analysis as well. The Gower distance metric is suitable for this purpose, as it can simultaneously handle both quantitative and qualitative variables.

Cluster analysis was done using Gower's distance.

2. Incorporating regional information into the clustering analysis will facilitate a clearer understanding of genetic flow of the accession

Passport data used in the analysis and discussion.
