# Supplementary material for: Population structure and diversity of common bean (Phaseolus vulgaris L.) landraces in the Peruvian Amazon
Source: PLoS One. 2026 Jul 20;21(7):e0332680. doi: 10.1371/journal.pone.0332680 (PMC13384298; doi:10.1371/journal.pone.0332680)
Supplement: Attachment — Submitted filename: Reviewers_comments-answer RO.docx [file pone.0332680.s007.docx]

Journal Requirements:

1. Please ensure that your manuscript meets PLOS ONE's style requirements, including those for file naming. The PLOS ONE style templates can be found at…

The manuscript was adapted to the template

2. In your Methods section, please provide additional information regarding the permits you obtained for the work. Please ensure you have included the full name of the authority that approved the field site access and, if no permits were required, a brief statement explaining why.

It is included the file of permission on genetic resources accessions from Peruvian authority (INIA), which was not required to develop the research.

3. Thank you for stating the following in the Acknowledgments Section of your manuscript:

4. We note that Figures 1 and 11 in your submission contain [map/satellite] images which may be copyrighted. All PLOS content is published under the Creative Commons Attribution License (CC BY 4.0),…

The maps were elaborate using R program, according the following methodological approach for creating the map of beens accessions in Software R consisted of the following sequence: 1) obtaining elevation data (DEM) for the Peruvian territory using the get_elev_raster() function from the elevatr package; 2) loading political boundary vector layers via rnaturalearth; 3) importing and converting collected bean occurrence points—stored in clipboard data—into a spatial format (sf); and 4) integrating all layers into a two‑dimensional map with ggplot2, overlaying the shaded relief (elevation raster with a customized color palette), departmental borders, collection locations and cartographic elements such as a north arrow and scale bar. Finally, the map was exported as a TIF image.

**Additional Editor Comments:**

Since authors have genotypic data and phenotypic data on multiple traits, I suggest to run the GWAS to identify genomic regions associated with traits.
Figure quality need to be improved. increase font size in Fig 6, 7 and 12.

This GWAS analysis will be done for another paper which should include multilocal agronomic evaluation and nutritional analysis.

Figures quality were improved according to the journal recommendations.

Reviewers' comments:

**5. Review Comments to the Author**

Please use the space provided to explain your answers to the questions above. You may also include additional comments for the author, including concerns about dual publication, research ethics, or publication ethics. (Please upload your review as an attachment if it exceeds 20,000 characters)

Reviewer #1: Page 9: a. Kindly include the aim of study

It was added the aim: “For this reason, the main aim of this study was to assess the genetic diversity of *P. vulgaris* populations.”

b. I believe 667 accessions were collected but only 476 were analysed. Please correct this this number in the abstract, materials and method and result section.

The reason of discordance number, it was already indicated in page 9 (Morphological variability)

We describe the characterization of 476 accessions (from 667 sowed), the rest of 191 accessions did not progress in the field trial.

c. Kindly expand the result section to strengthen the abstract. The information on population structure and clustering is not clearly highlighted, and much of the rich detail appears to be hidden.
It was expanded.

Kindly refer to the attached manuscript for my additional comments. Thank you. Excellent write up.

Reviewer #2: The study entitled “Population structure and diversity of common bean (Phaseolus vulgaris L.) landraces in the Peruvian Amazon” provides interesting advances in understanding the population structure and genetic diversity of common bean landraces collected from different region by utilizing both morphological and SNP markers information. However, the manuscript, in its current form, requires substantial revision before it can be considered for publication, most especially in mythology, results presentation, and conclusion. Below is the specific point to be addressed:
1. The Materials and Methods section lack essential details. Critical information such as descriptions of the recorded traits and the procedures used for data processing and analysis were insufficiently described. To ensure the reproducibility of this study, please provide a detailed description of each studied trait, rather than only indicating the number of colors or types.

It was improved the explanation on methodology:

Data was recorded as following for each descriptor, taken into consideration common bean descriptors chart:

**Growth habit**: Determined based on the description of growth habits and reference diagrams in the descriptor.

**Days to flowering**: Number of days elapsed from sowing until the opening of the first floral bud in at least 50% of the evaluated plants.

**Color of standard**: Observation on freshly opened flowers from the inflorescence at the fourth node on each evaluated plant, according to the descriptor color chart.

**Wing color**: Observation on freshly opened flowers from the inflorescence at the fourth node on each evaluated plant, according to the descriptor color chart.

**Green pod color**: Observation on 10 fully developed immature pods, according to the descriptor color chart.

**Pod length**: Average length (cm) of 10 pods obtained from five plants.

**Pod cross-section**: Transverse section of 10 fully developed immature pods at the level of the second seed from the distal end, according to the descriptor description chart.

**Pod curvature**: Observation of 10 fully developed immature pods, according to the descriptor description chart.

**Number of locules**: Average of number of locules from longest pod on10 pods obtained from five plants.

**Pod wall fiber**: Observation on 10 pods obtained from five plants, according to the descriptor model.

**Pod apex position**: Observation of 10 pods obtained from five plants, according to the descriptor model.

**Pod apex orientation**: Observation of 10 pods obtained from five plants, according to the descriptor model.

**Pod color at the physiological matur**ity: Observation of 10 dry pods at the harvesting time, according to the descriptor color chart.

**Seed coat pattern**: Observation of 10 seeds, taken from the seed closest to the apex of each pod, according to the descriptor pattern chart.

**Seed coat lighter color**: Observation of primary (light) seed coat color on 10 seeds, taken from the seed closest to the apex of each pod, according to the descriptor color chart.

**Seed coat darker color**: Observation of secondary (background) seed coat color on 10 seeds, taken from the seed closest to the apex of each pod, according to the descriptor color chart.

**Seed brightness**: Observation of seed coat luster on10 seeds, taken from the seed closest to the apex of each pod.

Seed shape: Observation of 10 seeds, taken from the middle of each pod.

Prior to data analysis, it was estimated the average for the quantitative data and recorded the mode for the qualitative data (the value that appears most frequently in the dataset, representing the most common category of descriptors). Then, the matrix was used in the univariate and multivariate analysis.

…..
2. The Results section primarily restates elements of the methodology and discussion rather than presenting the main findings in detail, and the figure quality could be improved to allow proper evaluation

Figures’ quality was improved.

3. “Despite its importance, most studies on common bean in Peru have focused on commercial varieties grown in the highlands and coastal regions, emphasizing agronomic practices and genetic improvement using both local and introduced varieties”. Please provide some references that have investigated these areas.

There is no specific reference about the following paragraph, it was written taking into consideration our comprehensive bibliographic revision on common beans in Peru and our knowledge on research activities at the UNALM Legume Program, … “in Peru have focused on commercial varieties grown in the highlands and coastal regions, emphasizing agronomic practices and genetic improvement using both local and introduced varieties”

4. The conclusion provides only a summary of the results and does not effectively address the research objective

This article addresses the under characterization of genetic diversity in common bean (landraces from the Peruvian Amazon, which limits their use in breeding programs. Prior to this study, most research focused on commercial cultivars from highland and coastal regions, neglecting the unique genetic resources found in the Amazon. The comprehensive evaluation of 476 accessions using both phenotypic and genomic approaches found two major genetic groups corresponding to Andean and Mesoamerican gene pools, each with distinct subgroups. There was high allelic richness but low observed heterozygosity, consistent with the self-pollinating nature of the species. This study enhances our understanding of the genetic diversity present in Amazonian common beans, highlighting the need for conservation strategies to preserve these valuable resources for future breeding efforts.

5. “Cluster analysis based on morphological traits grouped the accessions into four major clusters

In was corrected.

6. (Figure 3).” But Figure 3 showed Boxplot of quantitative descriptors, please verify and indicate the correct figure

It was corrected; it is figure 5.

7. Although the data were said to be filtered at MAF of ≥ 1%, the MAF distribution extended to 0.5 (Figure 8E). Please clarify this discrepancy
It is 0.5%, already corrected.

In addition to the comment above, the following suggestion would enrich the manuscript
1. Since the study was conducted at a single location, in a non-replicated trial with no appropriate experimental design, the quantitative traits may be misleading (i.e., the observed differences might not reflect the true genetic merit of the landraces under investigation). Therefore, it is recommended to include qualitative descriptors in the clustering analysis as well. The Gower distance metric is suitable for this purpose, as it can simultaneously handle both quantitative and qualitative variables.

Cluster analysis was done using Gower’s distance.

2. Incorporating regional information into the clustering analysis will facilitate a clearer understanding of genetic flow of the accession

Passport data used in the analysis and discussion.
