# Supplementary material for: Population structure and diversity of common bean (Phaseolus vulgaris L.) landraces in the Peruvian Amazon
Source: PLoS One. 2026 Jul 20;21(7):e0332680. doi: 10.1371/journal.pone.0332680 (PMC13384298; doi:10.1371/journal.pone.0332680)
Supplement: Attachment — Submitted filename: Reviewer comments-answer.docx [file pone.0332680.s009.docx]

Reviewer #1: The author has done a good job reviewing the manuscript. However, some concerns were not addressed. Please refer to the attached manuscript.

The information on population structure and clustering is not clearly highlighted, and much of the rich detail appears to be hidden. Results should be presented with quantitative values and rather than as descriptive text alone.

The abstract was rewritten as follows:

Common bean (*Phaseolus vulgaris* L.) is a globally important grain legume, yet the genetic diversity of landraces from the Peruvian Amazon remains poorly characterized. This study aimed to assess the population structure and genetic diversity of Amazonian common bean by integrating morphological and genome-wide SNP data. A total of 476 accessions were phenotyped using 18 morphological traits, and 647 accessions were genotyped with 23,050 high-quality DArTseq SNP markers. Cluster and population structure analyses consistently identified two major genetic groups corresponding to the Andean (n = 284) and Mesoamerican (n = 363) gene pools, with further subdivision into four subgroups at K = 4. Principal Coordinate Analysis supported this structure, with the first axis explaining 62.62% of the molecular variation. Genetic diversity was high, with 94.9% of SNPs successfully mapped and more than 94% of loci polymorphic in both groups. However, observed heterozygosity was low (Ho = 0.035–0.041) compared to expected heterozygosity (He ≈ 0.147–0.148), and inbreeding coefficients were high (FIS = 0.52–0.54), consistent with the predominantly self-pollinating mating system. Hill number analyses indicated similar allelic richness between groups (q = 0: α ≈ 1.95–1.97) and low differentiation in allele presence (β = 1.04), whereas differentiation increased when allele frequencies were considered (q = 2: β ≈ 1.4–1.5). Morphological analyses revealed substantial phenotypic variation, including flowering time (32–75 days), pod length (6.5–17.3 cm), and number of locules per pod (2–11), and identified four distinct phenotypic clusters. No redundant accessions were detected. These results indicate that Amazonian landraces harbor high levels of genetic and phenotypic diversity structured by gene pool origin and local adaptation. The integration of morphological and SNP data provides a robust framework for germplasm characterization and highlights the importance of conserving these genetic resources for future breeding and sustainable agriculture.

The suggested inclusion of pod weight was not implemented.

Answer: Pod weight was not recorded.

How many individuals or accessions per group? Please state the quantitative value

It was added as follows:

Cluster analysis revealed the presence of four well-defined groups among common bean (*Phaseolus vulgaris* L.) accessions from the Peruvian Amazon (Fig. 5). Group sizes were uneven, comprising 78, 97, 250, and 51 accessions for clusters 1 through 4, respectively. This clear partitioning indicates a structured pattern of morphological diversity within these landraces, the number.

Kindly state how many accessions per group

It was added in the text as follows:

Clustering analysis based on the neighbor‐joining algorithm resolved the evaluated accessions into two well‐defined major groups, reflecting their underlying genetic relationships (Fig. 9A). The first cluster comprised 284 accessions assigned to the Andean gene pool, whereas the second included 363 accessions corresponding to the Mesoamerican gene pool. This clear bifurcation underscores the strong genetic differentiation between the two domestication lineages. The first group comprised primarily accessions collected from the Peruvian Amazon, together with reference accessions included as an outgroup of Andean origin from the Peruvian highlands, the coastal region, and CIAT.

Kindly ensure that results are not included within the discussion section. Please correct all through.

Below text moved from Discussion to Results Section

Of the Amazonia accessions, 56.1 % clustered with the Mesoamerican pool and 43.9 % with the Andean gene pools.

By comparison, when considering K = 4, both the Andean and Mesoamerican gene pool was clustered in two subgroups, with low admixture.

Kindly indicate the contributory role of David Saravia (DS)

It was corrected in the text as follows:

## **Author contributions**

….. contributed to investigation; DS, RR and CP to data curation, writing

Reviewer #2: The conclusion could be improved to address specific objectives and should be written in one paragraph

It was modified in the text as follows:

This study addresses the limited characterization of genetic diversity in common bean (Phaseolus vulgaris L.) landraces from the Peruvian Amazon, a region that has been largely overlooked in favor of highland and coastal germplasm. Our results show that these landraces harbor substantial morphological and genomic diversity, confirming the Amazon as an important reservoir of agrobiodiversity. Both morphological descriptors and SNP-based analyses consistently identified two major groups corresponding to the Andean and Mesoamerican gene pools, each further subdivided into distinct subgroups that include unique Amazonian accessions. High allelic richness combined with low observed heterozygosity was consistent with the predominantly self-pollinating nature of the species and the associated high levels of inbreeding, while genetic variation was primarily explained by differences in allele frequencies rather than the presence of unique alleles, indicating similar levels of internal diversity across groups. The absence of redundant accessions highlights the distinct genetic contribution of each sampled landrace. The combined use of morphological traits and DArTseq SNP markers proved effective for detecting population structure and diversity patterns, supporting their complementary role in germplasm characterization. Given that much of this diversity is maintained in situ by local farmers, these findings highlight the importance of strengthening both in situ and ex situ conservation strategies. Overall, this work provides a baseline for future research and supports the use of Amazonian common bean landraces as a valuable resource for breeding programs aimed at improving adaptation to tropical environments and resilience to changing climatic conditions.
